# eHealth Interventions to Address Sexual Health, Substance Use, and Mental Health Among Men Who Have Sex With Men: Systematic Review and Synthesis of Process Evaluations

**DOI:** 10.2196/22477

**Published:** 2021-04-23

**Authors:** Rebecca Meiksin, G J Melendez-Torres, Jane Falconer, T Charles Witzel, Peter Weatherburn, Chris Bonell

**Affiliations:** 1 London School of Hygiene & Tropical Medicine London United Kingdom; 2 Peninsula Technology Assessment Group College of Medicine and Health University of Exeter Exeter United Kingdom

**Keywords:** eHealth, digital health, men who have sex with men, sexual health, HIV, STI, substance use, mental health, systematic review, process evaluation

## Abstract

**Background:**

Men who have sex with men (MSM) face disproportionate risks concerning HIV and other sexually transmitted infections, substance use, and mental health. These outcomes constitute an interacting syndemic among MSM; interventions addressing all 3 together could have multiplicative effects. eHealth interventions can be accessed privately, and evidence from general populations suggests these can effectively address all 3 health outcomes. However, it is unclear how useable, accessible, or acceptable eHealth interventions are for MSM and what factors affect this.

**Objective:**

We undertook a systematic review of eHealth interventions addressing sexual risk, substance use, and common mental illnesses among MSM and synthesized evidence from process evaluations.

**Methods:**

We searched 19 databases, 3 trials registers, OpenGrey, and Google, and supplemented this by reference checks and requests to experts. Eligible reports were those that discussed eHealth interventions offering ongoing support to MSM aiming to prevent sexual risk, substance use, anxiety or depression; and assessed how intervention delivery or receipt varied with characteristics of interventions, providers, participants, or context. Reviewers screened citations on titles, abstracts, and then full text. Reviewers assessed quality of eligible studies, and extracted data on intervention, study characteristics, and process evaluation findings. The analysis used thematic synthesis.

**Results:**

A total of 12 reports, addressing 10 studies of 8 interventions, were eligible for process synthesis. Most addressed sexual risk alone or with other outcomes. Studies were assessed as medium and high reliability (reflecting the trustworthiness of overall findings) but tended to lack depth and breadth in terms of the process issues explored. Intervention acceptability was enhanced by ease of use; privacy protection; use of diverse media; opportunities for self-reflection and to gain knowledge and skills; and content that was clear, interactive, tailored, reflective of MSM’s experiences, and affirming of sexual-minority identity. Technical issues and interventions that were too long detracted from acceptability. Some evidence suggested that acceptability varied by race or ethnicity and educational level; findings on variation by socioeconomic status were mixed. No studies explored how intervention delivery or receipt varied by provider characteristics.

**Conclusions:**

Findings suggest that eHealth interventions targeting sexual risk, substance use, and mental health are acceptable for MSM across sociodemographic groups. We identified the factors shaping MSM’s receipt of such interventions, highlighting the importance of tailored content reflecting MSM’s experiences and of language affirming sexual-minority identities. Intervention developers can draw on these findings to increase the usability and acceptability of integrated eHealth interventions to address the syndemic of sexual risk, substance use, and mental ill health among MSM. Evaluators of these interventions can draw on our findings to plan evaluations that explore the factors shaping usability and acceptability.

## Introduction

Men who have sex with men (MSM) face disproportionate risks in relation to use of tobacco, alcohol, and legal and illegal drugs (henceforth termed *substance use*), mental ill health, and HIV and other sexually transmitted infections [1-12]. These outcomes constitute a syndemic, whereby they interact [2,13] to increase overall risks of substance use, mental ill health, and sexual risk among MSM across age groups and ethnicities [13-16]. This clustering and interaction of adverse outcomes suggests that interventions which address substance use, mental ill health, and sexual risk together could have multiplicative effects. eHealth interventions, delivered via electronic media and devices, offer a means to access prevention and treatment programs privately and anonymously particularly for MSM, who generally report high use of such media and devices [17]. Systematic reviews for general or mixed populations suggest that eHealth interventions can be effective in reducing alcohol use [18] and addressing common mental health issues [19-25], and emerging evidence indicates potential effects on drug use and sexual risk [26-29]. The few reviews assessing eHealth interventions among MSM suggest they can achieve short-term behavior change for sexually transmitted infections/HIV prevention [28,30], increase HIV testing [28,31], and improve treatment adherence among HIV-positive MSM [31,32]. To our knowledge, no systematic reviews have assessed the effectiveness of eHealth interventions to reduce substance use or improve mental health among MSM.

In addition to their effectiveness, it is important to examine what factors affect the usability and acceptability of eHealth interventions addressing these various outcomes among MSM. This should inform the development of interventions that can feasibly and acceptably address all 3 outcomes together [33]. Designing eHealth interventions to address MSM’s needs and affirm sexual-minority identities is likely to be important [34]. Product assessments suggest that eHealth interventions to reduce depression and anxiety among the general population rarely address the needs of gay and lesbian users [34]. However, there have been no systematic reviews to date conducted on the acceptability and usability of eHealth interventions addressing sexual health, substance use, or mental health risks among MSM.

Toward this end, we undertook a systematic review of eHealth interventions addressing these 3 outcomes and targeting this population. We included interventions addressing these outcomes together or separately, and aimed to synthesize evidence of effectiveness, describe intervention theories of change, and synthesize evidence from economic and process evaluations. This paper reports on the synthesis of process evaluations examining what factors related to interventions, providers, participants, or contexts (ie, environmental or structural factors) promote or impede delivery or receipt of these interventions.

## Methods

### Inclusion Criteria

Reports eligible for inclusion in the overall review reported on eHealth interventions that were delivered by mobile phone apps, the internet, or other electronic communication technology; offered ongoing support to populations consisting entirely or principally of gay, bisexual, and other men (including cisgender and transgender men) who have sex with men; and aimed to prevent HIV/sexually transmitted infections, sexual risk behavior, alcohol, tobacco or drug use, anxiety, or depression. We excluded interventions that offered one-off (rather than ongoing) support or that involved human providers (eg, in a chat room). Reports eligible for the process evaluation synthesis reported on characteristics of interventions, providers, participants, or context affecting delivery or receipt of eligible interventions. We included published and grey literature and set no restrictions by location or language.

### Search Strategy and Screening for Eligibility

Terms used in our search strategy covered 2 concepts joined by the Boolean operator “and”: MSM and eHealth. We searched 19 databases containing health and social science literature (from October 2018 to November 2018 and updated on April 2020). Our complete search strategy for the original OvidSP MEDLINE database is included in Multimedia Appendix 1 and the search strategies for all databases are available at the London School of Hygiene & Tropical Medicine’s Data Repository [35]. We conducted additional searches by searching 3 clinical trials registers, the OpenGrey database, and Google (limited to first 100 results), and by completing reference checks of included reports and requests from experts.

Citations were uploaded to EndNote (Clarivate Analytics), deduplicated, and then uploaded to EPPI-Reviewer (version 4.0, EPPI-Centre) for screening. CB and JF independently screened titles and abstracts in batches of the same 50 references, resolving disagreements by discussion. After reaching an agreement rate of at least 95%, they single-screened all remaining citations. Screening of full texts followed a comparable process.

### Data Extraction and Assessment of Quality

For process evaluations, CB and RM used an adapted version of an existing tool [36] to independently extract data reporting empirically on how processes of delivery or receipt varied with characteristics of interventions, providers, participants, or contexts. They also extracted data on basic study details, methods, and intervention descriptions. They then independently assessed the quality of process evaluation reports using standard Critical Appraisal Skills Program and EPPI-Centre tools [37]. These addressed the rigor of sampling, data collection and data analysis; the extent to which study findings were grounded in the data; whether the study privileged the perspectives of participants (eg, by including open-ended questions or otherwise allowing space for participants to set out their own views); and the breadth and depth of findings (ie, the extent to which the study explored a broad range of process issues or provided in-depth insights into participant perspectives). Drawing on these criteria, each reviewer then assigned weights (high, medium, or low) to rate the reliability or trustworthiness of the findings, and the usefulness of the findings for shedding light on the research question (ie, the extent to which they shed light on how processes of intervention delivery and receipt varied with characteristics of interventions, providers, participants, or contexts). Reliability reflected the trustworthiness of the overall findings (ie, the extent to which the methods employed were rigorous and could minimize bias and error in the findings). CB and RM met to compare their assessments, resolving all differences through discussion.

### Data Analysis

Using thematic synthesis methods [38-40], we explored themes concerning the characteristics of interventions, participants, and context acting as potential barriers and facilitators of delivery and receipt, and which themes applied across or only within the domains of sexual health, substance use, and mental health interventions. Synthesis followed a meta-ethnographic approach [41]. We undertook line-by-line coding of reports examining “first-order constructs” (directly quoted qualitative data) and second-order constructs (author interpretations). In the case of findings from quantitative study components, we coded author interpretations, first checking as part of quality assessment whether these aligned with quantitative data presented (ie, the extent to which study findings were grounded in the data, as noted above). Coding developed third-order constructs by drawing connections between these data. We did not exclude studies based on quality assessment, but rather gave less interpretive weight to conclusions that drew only on poorer-quality reports.

First, CB and RM prepared tables describing the quality of each evaluation, intervention details, study site and population, and pertinent findings. Second, the reviewers independently piloted coding of 2 high-quality studies. Coding began with in vivo codes which closely reflected the words used in the findings sections. The reviewers then grouped and organized codes, applying axial codes that reflected higher-order, cross-cutting themes. They then met to compare and contrast their coding, developing an overall set of codes. Next, they each went on to independently code the remaining reports, drawing on the agreed set of codes and developing new in vivo and axial codes as new themes emerged. At the end of this process, they met to compare their sets of codes. They identified commonalities, differences of emphasis, and contradictions to develop an overall analysis which drew on the strengths of the 2 sets of codes and which resolved any contradictions or inconsistencies.

## Results

### Results of the Search for Overall Review

**Figure 1:**
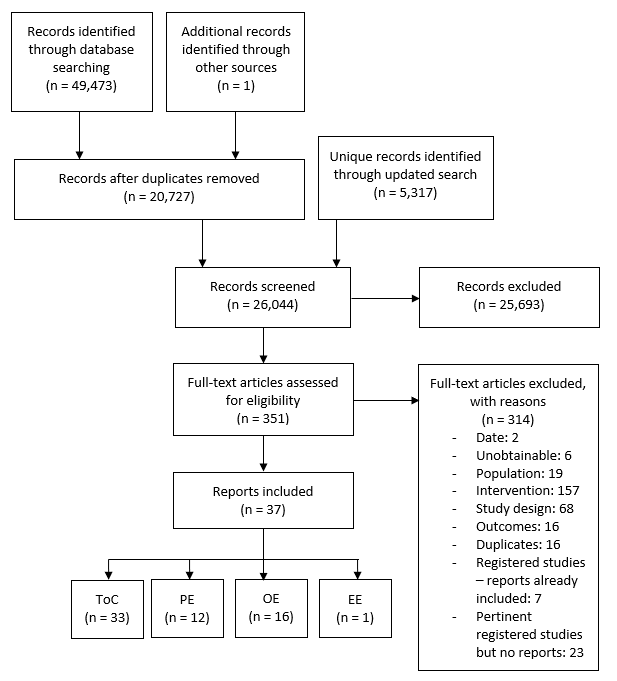
Searches and screening. EE: economic evaluation; OE: outcome evaluation; PE: process evaluation; ToC: theory of change.

Our search retrieved 26,044 unique results, including 1 identified via reference checking (see Figure 1). After title and abstract screening, 6 full texts were unobtainable and 345 reports were screened on full text. Of these, 37 reports were eligible for inclusion in the overall review. These reported on 28 unique studies and 23 unique interventions [42-78]. Reports were published between 2006 and 2020, with most published in 2015 or later.

### Reports Included in Process Evaluation Synthesis

Twelve reports were eligible for inclusion in the process evaluation synthesis (see Multimedia Appendix 2 for details of each intervention and Multimedia Appendix 3 for study characteristics) [42,49,52,54,59,63,64,67,68,75,76,78]. These reported on 11 studies which assessed 8 unique interventions, and 2 interventions were assessed in 2 different studies [54,59,67,78]. Included process evaluation reports presented findings on how intervention receipt (but not delivery) varied by characteristics of the intervention [42,49,52,59,63,64,67,68,75,76,78] participants [42,49,59,63,64,75,76,78] and context [54,75,78] but not providers. Additionally, 3 interventions addressed sexual health alone [54,68,75,78], 2 addressed mental health alone [42,63,64], 1 addressed sexual health and substance use [49,59,67], and 2 addressed all 3 outcomes of interest for this review [52,76] (see Multimedia Appendix 2). Moreover, 4 interventions targeted sexual minority youth or young adults [49,52,59,63,64,67,68], 2 targeted MSM more generally [42,75], 1 targeted rural MSM [54,78], and 1 targeted people living with HIV [76]. In terms of delivery mode, 5 were delivered via the internet [42,49,52,54,59,67,68,78], 2 via smartphone apps [75,76], and 1 via computer CD-ROM [63,64]. Process evaluations for 7 of the included interventions took place in the United States [42,49,52,54,59,67,68,75,76,78], and 1 took place in New Zealand [63,64,76].

### Quality Assessment

Multimedia Appendix 3 shows the results of our quality assessments. In total, 11 of the 12 included reports were assessed as reporting findings that were grounded in the data presented. Overall quality varied, with most reports assessed as medium or high quality. In terms of the reliability or trustworthiness of their overall findings, 4 reports were assessed as medium quality [42,68,75,76] and 8 as high quality [49,52,54,59,63,64,67,78]. In terms of their overall usefulness for addressing our research questions, 4 were assessed as low quality [52,54,68,78], 3 as medium quality [63,64,67], and 5 as high quality [42,49,59,75,76]. Only 1 report was assessed as high quality in both reliability/trustworthiness and usefulness [49,59], and all were assessed as medium or high quality in at least one of these 2 domains [42,49,52,54,59,63,64,67,68,75,76,78].

### Themes Emerging From Synthesis of Process Evaluation Reports

Multimedia Appendix 4 shows the relationship between primary, secondary, and tertiary codes developed through our analysis and synthesis of process data.

#### Intervention Characteristics Affecting Intervention Receipt

Nearly all process evaluations explored ways in which intervention characteristics affected receipt, although the included reports tended to lack breadth in the areas explored and in-depth exploration of the findings that they did report [42,49,52,59,63,64,67,68,75,76,78]. Nonetheless, several subthemes emerged in our analysis.

#### Ease of Use

Across health domains, acceptability was enhanced when interventions were easy to use and free of technical problems. Few technical problems were reported. For example, from studies assessed as medium reliability, 10% or fewer Smartphone Self-Monitoring users reported technical difficulties [76] and participants reported that the HealthMindr app was easy to use [75]. However, from studies of medium [42,76] and high [49,54,59,63,67,78] reliability, when participants did encounter technical issues, such as freezing [59] or incompatibility with mobile devices [42,49], this eroded acceptability. In a 2007 study of an intervention targeting rural MSM, features such as sound, animation or graphics could cause the intervention to load too slowly for participants with slower internet speeds, which authors suggested might derail participation [54].

From studies of medium and high reliability, accompanying materials outside of the electronic environment (such as printable materials [42] or a notebook [63,64]) were reported to potentially enhance acceptability, but participants disliked exercises that required using materials they might not have readily at hand [42].

#### Intervention Content

##### Clear and Comprehensive Content

From studies of medium reliability across health domains, it was apparent that intervention content which involved clear and comprehensive information facilitated acceptability. For example, Queer Sex Ed participants appreciated that this intervention provided comprehensive information on a range of sexual health and relationship topics rather than focusing narrowly on sexually transmitted infections [68]. In studies of other interventions, acceptability was reportedly enhanced where content was clear and up to date [42], while content participants found confusing detracted from acceptability [76].

##### Engaging Intervention Content

Fun [68] and enjoyable [42] content increased acceptability, and the use of different types of content arose as a common theme influencing the acceptability. For example, in studies of medium [42] and high [49,59,63,67] reliability, participants tended to give positive feedback on the use of diverse contents [42,49,59] including animations, videos, graphics, and games [67] as well as on the interventions’ visual appearances [43,63]. In a high-reliability study of Rainbow SPARX, users were particularly positive about the computer game format and the intervention’s “look and feel” [63] as expressed by one user aged 13 years: “I liked, like, how it looked really shiny on my computer, and it looked like a completely different world” [13]. Rainbow SPARX participants also liked particular characters who appeared in the game [63], a theme echoed in a high-reliability study of the Keep it Up! intervention where participants reported liking the scenarios and examples presented [67]. Factors detracting from acceptability included content that participants found boring, repetitive [42,76], too easy [63], too difficult or draining [42], “not soothing” [42], “cheesy” [49,59], or generally unenjoyable [42]; and videos that users judged as too long or that featured low-quality sound and dialogue [49].

##### Language and Tone

Language and tone emerged as an important aspect of acceptability across interventions addressing all 3 health domains and in studies of medium [42,68,75] and high [49,59,63,64,67] reliability. Participants liked what authors described as a “frank, candid, and sex-positive tone” [59], colloquial language [67], and what one participant described as an “up-beat manner” [67]. For example, Queer Sex Ed users appreciated that the intervention did not rely on “scare tactics” and that its content was easy to understand without making them feel “talked down to” [68]. A Keep it Up! user echoed this sentiment, describing the intervention as “realistic and not condescending or out of touch” [49].

There were also some challenges in getting the language right for MSM-specific interventions. Some users of the Rainbow SPARX and Online Mindfulness-Based Cognitive Therapy interventions suggested that the sexuality-related terminology could be improved [63] and voiced concerns about the intervention’s approach to sexual minorities and a feeling of “anti-gay sentiment”[42]. Avellar [42] suggests content might have been overly clinical and miscommunicated the aim of improving overall well-being, although it was not clear whether participant concerns stemmed primarily from intervention content or from content about participating in a research study.

##### Interaction and Personalization

Participants in studies of medium [68] and high [49,63,67] reliability valued interactive aspects of interventions spanning all 3 health outcomes. Studies of medium [75,76] and high [59] reliability found that individual-level tailoring based on participant assessments could enhance acceptability. For example, 81% of HealthMindr users found recommendations based on their responses useful or very useful [75] and Smartphone Self-Monitoring users recommended adding what the authors summarized as “more in-depth questions to better reflect their experiences” [76].

##### Privacy and Intrusiveness

In studies of medium reliability, privacy and intrusiveness emerged as important themes influencing acceptability across 2 interventions, which between them addressed all 3 health outcomes [75,76]. Some Smartphone Self-Monitoring users felt the intervention’s use of daily surveys on substance use, sexual behaviors, and medication adherence, and 4-times daily surveys on physical and mental health-related quality of life were too long or too frequent [76]. Users expressed concerns about privacy regarding questions assessing sexual behavior including experiences with individual partners [76]. The vast majority of HealthMindr app users (86%) reported feeling confident in the app’s security, including its password features and the fact that the app’s name and icon did not suggest it was focused on HIV prevention [75]. At least one participant in the Smartphone Self-Monitoring intervention was uncomfortable with geolocation tagging of phone survey responses, although the authors noted that participants were instructed on how to disable this feature [76].

##### Pacing and Structuring

The pacing and structuring of content influenced acceptability across health domains. In studies of medium [42] and high [49,64,67] reliability, a modular as opposed to single-session approach could reportedly help users absorb content [67] although they tended to like setting their own pace [64], and one suggested they would have preferred to complete all modules in one sitting [49]. Requiring a full week between sessions of the Online Mindfulness-Based Cognitive Therapy was reported as too long, detracting from acceptability [42].

Users liked when intervention content progressed in a cumulative way [42]. Module order and how far the participant had progressed could also affect acceptability. Findings from a high-reliability study of the 3-module Hope Project (addressing knowledge, motivation, and behavior), which randomized the order in which modules were delivered, suggested that participants were more likely to find the knowledge module interesting when they encountered it last rather than first [78]. Assessing level of interest after each module, this study also found that among those completing all modules, participants were more likely to report finding them very interesting after completing all 3 compared to only the first.

Program length arose as a common theme affecting the acceptability of some modular interventions. Users of the 8-session Online Mindfulness-Based Cognitive Therapy [42], the 7-module Keep it Up! intervention [49,59,67], and 5 five-module Queer Sex Ed intervention [68] suggested that these programs were too long or too time-consuming.

##### Content Designed To Be Relevant to Participants’ Lives and Experiences

Participants valued that interventions were designed for people like them. From studies of high reliability, it was apparent that participants valued when interventions presented realistic scenarios and examples and addressed issues relevant to their own lives [49,59,63,67]. A Keep it Up! user appreciated that the intervention “was geared towards gay men and it understood how we operate and how dating works in the contemporary moment” [49].

Users of the Rainbow SPARX and Queer Sex Ed interventions liked that these programs were “[lesbian, gay, bisexual, and transgender] LGBT–specific” [68], designed for young people [64], and included “’rainbow content” tailored to this group [63]. Some felt there was room to go further [63,68], for example by removing content on female sexual anatomy for MSM users and adding more trans-specific content [68].

Online Mindfulness-Based Cognitive Therapy users had mixed views on how effectively this intervention was tailored for people like them [42]. Some reported appreciating that the program was designed for men who were attracted to men, while others felt the intervention “did not have much value in the context of their lives” [42]. Some Rainbow SPARX users reported that tailoring could be further enhanced by including more sexuality-specific content [63].

#### Perceived Usefulness of the Intervention

##### Gaining Knowledge and Skills

In studies of medium [42] and high [49,52,59,63,64,68,78] reliability, participants frequently cited perceived usefulness as positive in terms of the knowledge and skills the interventions aimed to impact. For example, Queer Sex Ed users liked that the intervention aimed to support communication and closeness with their partners, helping, as one participant described, to “open up doors to healthy communication” [68].

##### Opportunities for Self-Monitoring and Self-Reflection

Findings from the evaluation of the Smartphone Self-Monitoring intervention (targeting sexual health, substance use, and mental health outcomes) suggest that some participants valued its daily, mobile-based self-monitoring in contrast to the comparison group’s biweekly, web-based approach. One user described the benefits this way [76]:

Helps me keep a “log”, like therapy—but can do it every day instead of waiting for a week to see your therapist…Nice to do it throughout the day, multiple times a day, on a daily basis. Life happens daily—not weekly like when you see a therapist.

Participants in 3 interventions, which between them addressed all 3 health domains, highlighted the opportunities for introspection and self-reflection that the interventions presented [42,49,59,67,76]. For instance, a Smartphone Self-Monitoring user described the following [76]:

I started changing my behavior once I started taking the surveys—I have been thinking about it for a while but the surveys make me concentrate on certain areas of my life that I wasn’t focusing on.

A few also reported that engaging in self-monitoring across multiple domains enhanced their awareness of the relationships between their substance use, sexual behaviors, and other triggers for drug use [76]. A Keep it Up! user described how observing the characters in the intervention helped him to reflect on his own behaviors [67]:

I was able to see mistakes that I make in the actions of the characters. I wasn’t completely aware of my behavior until I judged a character’s behavior and then compared the same behavior to my own.

##### Opportunity for Self-Expression

Participants in the Smartphone Self-Monitoring intervention, which addressed all 3 health outcomes, valued the opportunity for self-expression that the intervention offered, as described by this participant: “I feel free to vent to the phone about things that I can’t talk to my partner about—I can really express how I feel” [76].

#### Participant Characteristics Affecting Intervention Engagement and Receipt

Evaluations of 4 interventions (2 targeting sexual health alone [75,78], 1 targeting mental health alone [42], and 1 targeting sexual health and substance use [49]) quantitatively explored the relationship between participant characteristics and intervention engagement.

A medium-reliability study of the HealthMindr mobile phone app found no differences in the time spent on the app by participant location in different cities in the United States, age, ethnicity, or knowledge of local HIV testing [75], while a high-reliability study of the Keep it Up! intervention, targeting young ethnically and racially diverse MSM, found that among Black users, those with graduate degrees spent more time on the intervention than those with high school–level or lower levels of education [49]. A study of medium reliability found no significant variation in retention for a modular mental health intervention by age, socioeconomic status, ethnicity, internalized homonegativity, or experience of homophobic bullying [42]. A study of high reliability found no differences in participants completing 1 versus all 3 modules of the Hope Project (an extension of the WRAPP intervention, targeting rural MSM) by age, ethnicity, marital status, sexual orientation, education, or student status, but did find higher completion among higher-earning participants [78].

Madkins et al [49] conducted a high-reliability, extensive exploration of the relationship between participant characteristics and receipt of the Keep it Up! intervention, which was developed with the engagement of diverse young MSM and designed for young MSM of all racial groups [67]. Researchers found several differences in the acceptability of the Keep it Up! intervention by race/ethnicity, education level, age, and city in the United States [49]. Black, Latino, and other non-White users reported higher acceptability in a range of domains than did White users, and Latino users rated content more highly compared to other non-White users. In the overall sample, users with high school–level education or lower rated the intervention more highly than those with higher education. Exploring the interaction of race/ethnicity and education level, the study found that higher levels of education were associated with lower acceptability among White users, while no such differences were found among Black, Latino, or other non-White users. Older users and those in Atlanta compared to New York tended to rate modules more highly.

Exploring intervention receipt qualitatively, a high-reliability study found that for Rainbow SPARX, a computer game intervention for sexual minority youth aged 13-19 years, some older users reported that some aspects were too easy and the program “babied” them [63]. Acknowledging the challenge of designing a program appropriate for a range of young people, one participant aged 19 years made the following remark [63]:

I thought some things were a little easy…Like overall it wasn’t difficult to figure out what you needed to do. Those little puzzles were quite easy to do. I guess it would be hard to make them more difficult though because you would have to be careful that everyone could actually get it.”

Qualitative research with participants of Rainbow SPARX and Smartphone Self-Monitoring found that these interventions could play a role in complementing the external mental health support participants were receiving [64,76].

#### Contextual Factors Affecting Intervention Engagement

Few studies explored how the context for using the intervention was associated with the experience of its use. Those that did focused on internet speed in the high-reliability 2007 [54] and 2010 [78] studies of 2 iterations of the WRAPP sexual health intervention, which targeted rural MSM in the United States. Bowen, et al [54] found that users with dial-up compared to high-speed connections were more likely to report taking too long to load program graphics, while Williams et al [78] found no differences in participants completing 1 versus all 3 modules by type of internet connection.

## Discussion

### Summary of Findings

One-third of reports included in the overall review included process evaluation data. All but one process evaluation took place in the United States. Most interventions targeted a single health domain of interest for this review (sexual health, substance use, or mental health), with the majority focused on sexual health. However, 2 aimed to address aspects of all 3 [52,76]. Some interventions employed personal tailoring, an approach that has been associated with effective eHealth behavior change interventions [79,80].

Process evaluations rarely explored how intervention receipt varied between contexts. We found no eligible reports examining what factors affected intervention delivery as opposed to receipt. This seems to reflect the emerging state of process evaluations in eHealth literature, with other reviews of eHealth interventions reporting a similar pattern [81-84]. There was some suggestion that slower internet speed could reduce acceptability of a multimedia intervention among rural MSM in the United States, who are less likely than nonrural residents to have high-speed internet at home [85].

In terms of intervention characteristics, as with use of eHealth interventions among general populations [83], participants appreciated when interventions were easy to use and free of technical problems, while incompatibility with mobile platforms detracted from acceptability and could impede participation. Privacy also emerged as an important aspect of acceptability, suggesting that detailed partner-level questions on sexual behavior could feel intrusive and that features protecting app access and obscuring the purpose of apps (for sensitive health domains) promote acceptability. The importance of privacy is also supported by existing evidence on behavior change interventions for MSM [86] and general populations [83].

Participants liked content that was interactive and aesthetically pleasing, and they enjoyed the use of diverse media such as animations, videos, and graphics. However, among rural MSM these media could also reduce loading times for users with slower internet connectivity. Although modular approaches could support users to absorb program content cumulatively, interventions that were too long detracted from acceptability, with some users preferring that less or no time be required between sessions. The ideal number and length of modules is likely dependent on a variety of participant, intervention, and contextual factors.

Individual tailoring based on participant characteristics and risk profiles increased acceptability, highlighting this as a particularly promising approach and aligning with other studies of eHealth behavioral interventions [79,84,87]. Participants valued when interventions presented scenarios and other content that reflected their experiences as MSM, an approach that stands in contrast to most existing eHealth interventions targeting mental health and HIV prevention [34,88]. Where interventions targeted sexual minority groups more broadly, some suggested further tailoring based on the sexual and gender identities of its users. The language and tone of intervention content emerged as an important factor shaping acceptability for MSM, who appreciated the use of colloquial, direct, “up-beat” [67], and sex-positive language. Our findings also highlight the importance of paying careful attention to language and framing to ensure that these affirm sexual-minority identities. That these concerns arose in interventions designed explicitly for sexual minority users, including one adapted for sexual minority young people using participatory approaches [63], suggests this is an important area to explore during the pilot phase of intervention development.

As with studies of eHealth interventions for general populations [81,83], perceived usefulness was key to acceptability. Participants liked gaining new knowledge and skills from eHealth interventions and developing an awareness of the relationship between sexual behaviors and substance use.

Although reviews of eHealth interventions for general populations report higher use and engagement among participants with higher levels of education [81,83,84], our findings suggest that in the context of generally high use of electronic devices among MSM [17], the targeting of intervention content might be a more important determinant of the relationship between education level and receipt of eHealth interventions than their electronic mode of delivery [49]. Similarly, our findings on the higher acceptability of the Keep it Up! intervention among Black, Latino, and other non-White users compared to White users suggest that eHealth interventions can be developed to enhance inclusive acceptability among racially and ethnically diverse users [49]. There was otherwise little evidence of engagement varying by sociodemographic factors, although findings on socioeconomic status were mixed [42,78]. Qualitative data suggest eHealth interventions can play a role in complementing external mental health support among MSM [63,76] and that interventions targeting all adolescents might struggle to pitch content appropriately for those across this age range [63].

### Limitations

Our process evaluation synthesis was limited by the size and quality of eligible reports. Most were assessed as medium- or high-quality in terms of their reliability and usefulness. However, studies often lacked depth and breadth of analysis, and only around half were judged to privilege MSM’s perspectives.

The vast majority of interventions targeted MSM only and all were evaluated principally among MSM, although 3 were assessed among samples that included cisgender women [63,64,68,76]. Author narratives and quantitative data did not always disaggregate MSM from other participants, presenting the possibility that specific findings from these 3 studies might reflect data from other groups. The process evaluation of Smartphone Self-Monitoring was the sole study contributing to findings on intervention benefits of self-monitoring and self-expression [76]. Although the intervention targeted people of all genders and sexual identities living with HIV, more than 80% of study participants identified as male and more than 80% identified as gay or bisexual [76]. In 2 studies, just under half of participants identified as female [63,64,68], but all themes informed by these studies also drew on other studies. The make-up of participants in these 3 studies is therefore unlikely to affect the validity of the themes to which they contributed. Studies of relevant interventions among broader sexual and gender minority populations might add further insight but could not be included, as we could not be certain which findings reflected experiences of or relevant to MSM.

### Implications for Research and Practice

eHealth interventions offer an avenue for MSM to access behavior change interventions privately, anonymously, and at times they find convenient. This synthesis identified several factors shaping MSM’s receipt of eHealth interventions addressing substance use, mental ill health, and sexual risk. Its findings suggest such interventions are acceptable for MSM across sociodemographic groups, although evidence in this area is limited and mixed. Different content for younger and older adolescents might be warranted. Variation in engagement and acceptability by participant characteristics should be explored in future research, and new interventions should be rigorously piloted to refine aspects affecting usability and acceptability [30,81].

Our review has identified several intervention characteristics affecting acceptability that existing research suggests are applicable to eHealth interventions for MSM and non-MSM populations alike. These include aspects of usability, length, aesthetics, multimedia use, and tailoring to participants’ personal and risk characteristics [79,81,83,84,86,87]. Other factors should be considered carefully in designing interventions for MSM, including ensuring that language and tone are affirming of sexual minority identity and that content reflects the reality and experiences of MSM. These findings can inform the development of integrated eHealth interventions to address the syndemic of substance use, mental ill health, and sexual risk among MSM and guide research questions for pilot and process evaluation studies. Going forward, process evaluations should explore a broader range of individual, intervention, and contextual factors that might affect implementation, and they should collect more in-depth—ideally qualitative—data privileging the perspectives of intended beneficiaries. Outcome evaluations of such eHealth interventions should conduct linked process evaluations wherever possible, which would shed further light on factors affecting how they are delivered and received [89].

Our findings regarding the value that participants place on interventions that address the reality of their lives and the interrelationships between the different domains of health suggest that eHealth interventions simultaneously addressing sexual health, substance use, and mental health might be particularly acceptable. Our review of theories of change [90] suggests that interventions addressing these different outcomes may aim to exert impacts via common mechanisms of action, further adding to the potential for eHealth interventions targeting multiple outcomes together. Our next analyses will assess the potential effectiveness of eHealth interventions on these outcomes.

## Appendix

Multimedia Appendix 1 Search terms and strategy for the MEDLINE database.

Multimedia Appendix 2 Descriptions of interventions included in process synthesis.

Multimedia Appendix 3 Characteristics and quality of appraisal of process evaluations.

Multimedia Appendix 4 Coding structure for process evaluation synthesis.

## Abbreviations

LGBT: lesbian, gay, bisexual, and transgender
MSM: men who have sex with men
NIHR PHR: National Institute for Health Research Public Health Research Programme

## References

[1] Knight DA, Jarrett D (2015). Preventive health care for men who have sex with men. Am Fam Physician.

[2] Stall R, Friedman M, Catania J, Wolitski R, Stall R, Valdiserri R (2008). Interacting epidemics and gay men's health: a theory of syndemic production among urban gay men. Unequal Opportunity: Health Disparities Affecting Gay and Bisexual Men in the United States.

[3] Beyrer C, Baral SD, van Griensven F, Goodreau SM, Chariyalertsak S, Wirtz AL, Brookmeyer R (2012). Global epidemiology of HIV infection in men who have sex with men. Lancet.

[4] Aghaizu A, Brown AE, Nardone A, Gill ON, Delpech VC, and contributors (2013). HIV in the United Kingdom 2013 report. Public Health England.

[5] Guasp A (2015). Gay and bisexual men's health survey (2013). Stonewall.

[6] Buffin J, Roy A, Williams H, Winter A (2012). Part of the picture: lesbian, gay and bisexual people's alcohol and drug use in England (2009-2011). The Lesbian & Gay Foundation; University of Central Lancashire.

[7] Lee J, Griffin G K, Melvin C (2009). Tobacco use among sexual minorities in the USA, 1987 to May 2007: a systematic review. Tob Control.

[8] Vosburgh HW, Mansergh G, Sullivan PS, Purcell DW (2012). A review of the literature on event-level substance use and sexual risk behavior among men who have sex with men. AIDS Behav.

[9] Hickson F, Bonell C, Weatherburn P, Reid D (2010). Illicit drug use among men who have sex with men in England and Wales. Addiction Research & Theory.

[10] Glass Rachel, Hope Vivian D, Tanner Claire, Desai Monica (2017). 'Slamming' among men who have sex with men accessing general drug services, in response to Schmidt, AJ et al., 2016, Illicit drug use among gay and bisexual men in 44 cities: Findings from the European MSM Internet Survey (EMIS). Int J Drug Policy.

[11] King M, Semlyen J, Tai SS, Killaspy H, Osborn D, Popelyuk D, Nazareth I (2008). A systematic review of mental disorder, suicide, and deliberate self harm in lesbian, gay and bisexual people. BMC Psychiatry.

[12] McFall S (2012). Understanding Society: Findings 2012. Institute for Social and Economic Research, University of Essex.

[13] Friedman M, Stall R, Silvestre Anthony J, Wei Chongyi, Shoptaw Steve, Herrick Amy, Surkan Pamela J, Teplin Linda, Plankey Michael W (2015). Effects of syndemics on HIV viral load and medication adherence in the multicentre AIDS cohort study. AIDS.

[14] Halkitis PN, Kapadia F, Bub KL, Barton S, Moreira AD, Stults CB (2015). A longitudinal investigation of syndemic conditions among young gay, bisexual, and other MSM: the P18 Cohort Study. AIDS Behav.

[15] Halkitis PN, Kupprat SA, Hampton MB, Perez-Figueroa R, Kingdon M, Eddy JA, Ompad DC (2012). Evidence for a syndemic in aging HIV-positive gay, bisexual, and other MSM: implications for a holistic approach to prevention and healthcare. Nat Resour Model.

[16] Jie W, Ciyong L, Xueqing D, Hui W, Lingyao H (2012). A syndemic of psychosocial problems places the MSM (men who have sex with men) population at greater risk of HIV infection. PLoS One.

[17] Melendez-Torres GJ, Nye E, Bonell C (2015). Internet sex-seeking is inconsistently linked with sexual risk in men who have sex with men: systematic review of within-subjects comparisons. Sex. Health.

[18] Riper H, Blankers M, Hadiwijaya H, Cunningham J, Clarke S, Wiers R, Ebert D, Cuijpers P (2014). Effectiveness of guided and unguided low-intensity internet interventions for adult alcohol misuse: a meta-analysis. PLoS One.

[19] Andersson G, Cuijpers P (2009). Internet-based and other computerized psychological treatments for adult depression: a meta-analysis. Cogn Behav Ther.

[20] Andrews G, Cuijpers P, Craske MG, McEvoy P, Titov N (2010). Computer Therapy for the Anxiety and Depressive Disorders Is Effective, Acceptable and Practical Health Care: A Meta-Analysis. PLoS ONE.

[21] Arnberg FK, Linton SJ, Hultcrantz M, Heintz E, Jonsson U (2014). Internet-delivered psychological treatments for mood and anxiety disorders: a systematic review of their efficacy, safety, and cost-effectiveness. PLoS One.

[22] Păsărelu CR, Andersson G, Bergman Nordgren L, Dobrean A (2016). Internet-delivered transdiagnostic and tailored cognitive behavioral therapy for anxiety and depression: a systematic review and meta-analysis of randomized controlled trials. Cognitive Behaviour Therapy.

[23] Spijkerman M, Pots W, Bohlmeijer E (2016). Effectiveness of online mindfulness-based interventions in improving mental health: A review and meta-analysis of randomised controlled trials. Clin Psychol Rev.

[24] Christensen H, Batterham P, Calear A (2014). Online interventions for anxiety disorders. Curr Opin Psychiatry.

[25] Kaltenthaler E, Parry G, Beverley C, Ferriter M (2008). Computerised cognitive-behavioural therapy for depression: systematic review. Br J Psychiatry.

[26] Gabarron E, Wynn R (2016). Use of social media for sexual health promotion: a scoping review. Glob Health Action.

[27] L'Engle Kelly L, Mangone ER, Parcesepe AM, Agarwal S, Ippoliti NB (2016). Mobile phone interventions for adolescent sexual and reproductive health: a systematic review. Pediatrics.

[28] Schnall R, Travers J, Rojas M, Carballo-Diéguez Alex (2014). eHealth interventions for HIV prevention in high-risk men who have sex with men: a systematic review. J Med Internet Res.

[29] Noar S, Black H, Pierce L (2009). Efficacy of computer technology-based HIV prevention interventions: a meta-analysis. AIDS.

[30] Nguyen LH, Tran BX, Rocha LEC, Nguyen HLT, Yang C, Latkin CA, Thorson A, Strömdahl Susanne (2019). A systematic review of eHealth interventions addressing HIV/STI prevention among men who have sex with men. AIDS Behav.

[31] Purnomo J, Coote K, Mao L, Fan L, Gold J, Ahmad R, Zhang L (2018). Using eHealth to engage and retain priority populations in the HIV treatment and care cascade in the Asia-Pacific region: a systematic review of literature. BMC Infect Dis.

[32] Muessig K, LeGrand S, Horvath K, Bauermeister J, Hightow-Weidman L (2017). Recent mHealth interventions to support medication adherence among HIV-positive men who have sex with men. Curr Opin HIV AIDS.

[33] Glasgow RE, Vogt TM, Boles SM (1999). Evaluating the public health impact of health promotion interventions: the RE-AIM framework. Am J Public Health.

[34] Rozbroj T, Lyons A, Pitts M, Mitchell A, Christensen H (2014). Assessing the applicability of e-therapies for depression, anxiety, and other mood disorders among lesbians and gay men: analysis of 24 web- and mobile phone-based self-help interventions. J Med Internet Res.

[35] Falconer J (2020). Search strategies for: how can e-health interventions reduce the ‘syndemic’ of HIV/STIs and sexual risk, substance use and mental ill health among men who have sex with men? [Data Collection]. London School of Hygiene & Tropical Medicine.

[36] Egan M, Bambra C, Petticrew M, Whitehead M (2009). Reviewing evidence on complex social interventions: appraising implementation in systematic reviews of the health effects of organisational-level workplace interventions. J Epidemiol Community Health.

[37] Shepherd J, Harden A, Rees R, Brunton G, Garcia J, Oliver S, Oakley A (2006). Young people and healthy eating: a systematic review of research on barriers and facilitators. Health Educ Res.

[38] Arai L (2005). It might work in Oklahoma but will it work in Oakhampton? Context and implementation in the effectiveness literature on domestic smoke detectors. Injury Prevention.

[39] Noyes J, Popay J, Garner P (2005). What can qualitative research contribute to a Cochrane systematic review of DOT for promoting adherence to tuberculosis treatment?.

[40] Thomas J, Harden A (2008). Methods for the thematic synthesis of qualitative research in systematic reviews. BMC Med Res Methodol.

[41] Green J, Torogood N (2018). Qualitative Methods for Health Research (4th edition).

[42] Avellar T (2016). The feasibility and acceptability of an online mindfulness-based cognitive therapy intervention for same-sex attracted men [Master's thesis]. University of California Santa Barbara. https://www.alexandria.ucsb.edu/lib/ark:/48907/f3ng4qs1.

[43] Cheng W, Xu H, Tang W, Zhong F, Meng G, Han Z, Zhao J (2019). Online HIV prevention intervention on condomless sex among men who have sex with men: a web-based randomized controlled trial. BMC Infect Dis.

[44] Chiou P, Liao P, Liu C, Hsu Y (2020). Effects of mobile health on HIV risk reduction for men who have sex with men. AIDS Care.

[45] Coulter RW, Sang JM, Louth-Marquez W, Henderson ER, Espelage D, Hunter SC, DeLucas M, Abebe KZ, Miller E, Morrill BA, Hieftje K, Friedman MS, Egan JE (2019). Pilot testing the feasibility of a game intervention aimed at improving help seeking and coping among sexual and gender minority youth: protocol for a randomized controlled trial. JMIR Res Protoc.

[46] Hirshfield S, Downing MJ, Chiasson MA, Yoon IS, Houang ST, Teran RA, Grov C, Sullivan PS, Gordon RJ, Hoover DR, Parsons JT (2019). Evaluation of Sex Positive! A video eHealth intervention for men living with HIV. AIDS Behav.

[47] Kuhns LM, Garofalo R, Hidalgo M, Hirshfield S, Pearson C, Bruce J, Batey DS, Radix A, Belkind U, Jia H, Schnall R (2020). A randomized controlled efficacy trial of an mHealth HIV prevention intervention for sexual minority young men: MyPEEPS mobile study protocol. BMC Public Health.

[48] Jones J, Dominguez K, Stephenson R, Stekler JD, Castel AD, Mena LA, Jenness SM, Siegler AJ, Sullivan PS (2020). A theoretically based mobile app to increase pre-exposure prophylaxis uptake among men who have sex with men: protocol for a randomized controlled trial. JMIR Res Protoc.

[49] Madkins K, Moskowitz D, Moran K, Dellucci T, Mustanski B (2019). Measuring aacceptability and engagement of the Keep It Up! internet-based HIV prevention randomized controlled trial for young men who have sex with men. AIDS Educ Prev.

[50] Reback CJ, Fletcher JB, Leibowitz AA (2019). Cost effectiveness of text messages to reduce methamphetamine use and HIV sexual risk behaviors among men who have sex with men. J Subst Abuse Treat.

[51] Tan RKJ, Koh WL, Le D, Tan A, Tyler A, Tan C, Banerjee S, Wong CS, Wong M, Chio MT, Chen MI (2020). Effect of a web drama video series on HIV and other sexually transmitted infection testing among gay, bisexual and queer men: study protocol for a community-based, pragmatic randomised controlled trial in Singapore: the People Like Us (PLU) Evaluation Study. BMJ Open.

[52] Bauermeister JA, Tingler RC, Demers M, Connochie D, Gillard G, Shaver J, Chavanduka T, Harper GW (2019). Acceptability and preliminary efficacy of an online HIV prevention intervention for single young men who have sex with men seeking partners online: the mydex project. AIDS Behav.

[53] Bauermeister JA, Tingler RC, Demers M, Harper GW (2017). Development of a tailored HIV prevention intervention for single young men who have sex with men who meet partners online: protocol for the mydex project. JMIR Res Protoc.

[54] Bowen AM, Horvath K, Williams ML (2007). A randomized control trial of Internet-delivered HIV prevention targeting rural MSM. Health Educ Res.

[55] Bowen AM, Williams ML, Daniel CM, Clayton S (2008). Internet based HIV prevention research targeting rural MSM: feasibility, acceptability, and preliminary efficacy. J Behav Med.

[56] Carpenter KM, Stoner SA, Mikko AN, Dhanak LP, Parsons JT (2010). Efficacy of a web-based intervention to reduce sexual risk in men who have sex with men. AIDS Behav.

[57] Christensen JL, Miller LC, Appleby PR, Corsbie-Massay C, Godoy CG, Marsella SC, Read SJ (2013). Reducing shame in a game that predicts HIV risk reduction for young adult men who have sex with men: a randomized trial delivered nationally over the web. Journal of the International AIDS Society.

[58] Davidovich U, de Wit J, Stroebe W (2006). Using the internet to reduce risk of HIV-infection in steady relationships: a randomized controlled trial of a tailored intervention for gay men. Utrecht University Repository.

[59] Greene GJ, Madkins K, Andrews K, Dispenza J, Mustanski B (2016). Implementation and evaluation of the Keep It Up! online HIV prevention intervention in a community-based setting. AIDS Educ Prev.

[60] Hirshfield S, Downing Martin J, Parsons JT, Grov C, Gordon RJ, Houang ST, Scheinmann R, Sullivan PS, Yoon IS, Anderson I, Chiasson MA (2016). Developing a video-based eHealth intervention for HIV-positive gay, bisexual, and other men who have sex with men: study protocol for a randomized controlled trial. JMIR Res Protoc.

[61] Kok G, Harterink P, Vriens P, Zwart O, Hospers H (2006). The gay cruise: developing a theory- and evidence-based Internet HIV-prevention intervention. Sex Res Soc Policy.

[62] Linnemayr S, MacCarthy S, Kim A, Giguere R, Carballo-Dieguez A, Barreras JL (2018). Behavioral economics-based incentives supported by mobile technology on HIV knowledge and testing frequency among Latino/a men who have sex with men and transgender women: protocol for a randomized pilot study to test intervention feasibility and acceptability. Trials.

[63] Lucassen MF, Hatcher S, Fleming TM, Stasiak K, Shepherd MJ, Merry SN (2015). A qualitative study of sexual minority young people's experiences of computerised therapy for depression. Australas Psychiatry.

[64] Lucassen MF, Merry SN, Hatcher S, Frampton CM (2015). Rainbow SPARX: a novel approach to addressing depression in sexual minority youth. Cognitive and Behavioral Practice.

[65] Milam J, Morris Sheldon, Jain Sonia, Sun Xiaoying, Dubé Michael P, Daar Eric S, Jimenez Gustavo, Haubrich Richard, CCTG 592 Team (2014). Controlled trial of an internet-based risk reduction intervention in HIV+ men who have sex with men.

[66] Milam J, Morris S, Jain S, Sun X, Dubé Michael P, Daar ES, Jimenez G, Haubrich R, CCTG 592 Team (2016). Randomized controlled trial of an internet application to reduce HIV transmission behavior among HIV infected men who have sex with men. AIDS Behav.

[67] Mustanski B, Garofalo R, Monahan C, Gratzer B, Andrews R (2013). Feasibility, acceptability, and preliminary efficacy of an online HIV prevention program for diverse young men who have sex with men: the keep it up! intervention. AIDS Behav.

[68] Mustanski B, Greene GJ, Ryan D, Whitton SW (2015). Feasibility, acceptability, and initial efficacy of an online sexual health promotion program for LGBT youth: the Queer Sex Ed intervention. J Sex Res.

[69] Mustanski B, Madkins K, Greene GJ, Parsons JT, Johnson BA, Sullivan P, Bass M, Abel R (2017). Internet-based HIV prevention with at-home sexually transmitted infection testing for young men having sex with men: study protocol of a randomized controlled trial of Keep It Up! 2.0. JMIR Res Protoc.

[70] Mustanski B, Parsons JT, Sullivan PS, Madkins K, Rosenberg E, Swann G (2018). Biomedical and behavioral outcomes of Keep It Up!: an eHealth HIV prevention program RCT. Am J Prev Med.

[71] Reback C, Fletcher J, Swendeman D (2017). Theory-based text messages reduce methamphetamine use and HIV sexual risk behaviors among MSM.

[72] Reback CJ, Fletcher JB, Swendeman DA, Metzner M (2019). Theory-based text-messaging to reduce methamphetamine use and HIV sexual risk behaviors among men who have sex with men: automated unidirectional delivery outperforms bidirectional peer interactive delivery. AIDS Behav.

[73] Rosser B, Oakes J, Konstan J, Hooper Simon, Horvath Keith J, Danilenko Gene P, Nygaard Katherine E, Smolenski Derek J (2010). Reducing HIV risk behavior of men who have sex with men through persuasive computing: results of the Men's INTernet Study-II. AIDS.

[74] Schonnesson LN, Bowen AM, Williams ML (2016). Project SMART: preliminary results from a test of the efficacy of a Swedish internet-based HIV risk-reduction intervention for men who have sex with men. Arch Sex Behav.

[75] Sullivan PS, Driggers R, Stekler JD, Siegler A, Goldenberg T, McDougal SJ, Caucutt J, Jones J, Stephenson R (2017). Usability and acceptability of a mobile comprehensive HIV prevention app for men who have sex with men: a pilot study. JMIR Mhealth Uhealth.

[76] Swendeman D, Ramanathan C, Baetscher L, Medich Melissa, Scheffler Aaron, Comulada W Scott, Estrin Deborah (2015). Smartphone self-monitoring to support self-management among people living with HIV: perceived benefits and theory of change from a mixed-methods randomized pilot study. J Acquir Immune Defic Syndr.

[77] Wilkerson JM, Danilenko GP, Smolenski DJ, Myer BB, Rosser BRS (2011). The role of critical self-reflection of assumptions in an online HIV intervention for men who have sex with men. AIDS Educ Prev.

[78] Williams M, Bowen A, Ei S (2010). An evaluation of the experiences of rural MSM who accessed an online HIV/AIDS health promotion intervention. Health Promot Pract.

[79] Morrison LG, Yardley L, Powell J, Michie S (2012). What design features are used in effective e-health interventions? A review using techniques from Critical Interpretive Synthesis. Telemed J E Health.

[80] Noar S, Black H, Pierce L (2009). Efficacy of computer technology-based HIV prevention interventions: a meta-analysis. AIDS.

[81] Chib A, Lin SH (2018). Theoretical advancements in mHealth: a systematic review of mobile apps. J Health Commun.

[82] Christie HL, Bartels SL, Boots LM, Tange HJ, Verhey FR, de Vugt ME (2018). A systematic review on the implementation of eHealth interventions for informal caregivers of people with dementia. Internet Interventions.

[83] Simblett S, Greer B, Matcham F, Curtis H, Polhemus A, Ferrão José, Gamble P, Wykes T (2018). Barriers to and facilitators of engagement with remote measurement technology for managing health: systematic review and content analysis of findings. J Med Internet Res.

[84] Perski O, Blandford A, West R, Michie S (2016). Conceptualising engagement with digital behaviour change interventions: a systematic review using principles from critical interpretive synthesis. Behav. Med. Pract. Policy Res.

[85] (2019). Internet/broadband fact sheet. Pew Research Center. Internet & Technology Web site.

[86] Hergenrather KC, Emmanuel D, Durant S, Rhodes SD (2016). Enhancing HIV prevention among young men who have sex with men: a systematic review of HIV behavioral interventions for young gay and bisexual men. AIDS Educ Prev.

[87] Gorini A, Mazzocco K, Triberti S, Sebri V, Savioni L, Pravettoni G (2018). A P5 approach to m-Health: design suggestions for advanced mobile health technology. Front Psychol.

[88] Sullivan PS, Jones J, Kishore N, Stephenson R (2015). The roles of technology in primary HIV prevention for men who have sex with men. Curr HIV/AIDS Rep.

[89] Moore GF, Audrey S, Barker M, Bond L, Bonell C, Hardeman W, Moore L, O'Cathain A, Tinati T, Wight D, Baird J (2015). Process evaluation of complex interventions: Medical Research Council guidance. BMJ.

[90] Meiksin Rebecca, Melendez-Torres G J, Falconer Jane, Witzel T Charles, Weatherburn Peter, Bonell Chris (2021). Theories of change for e-health interventions targeting HIV/STIs and sexual risk, substance use and mental ill health amongst men who have sex with men: systematic review and synthesis. Syst Rev.

