# Supplementary material for: eHealth Interventions to Address Sexual Health, Substance Use, and Mental Health Among Men Who Have Sex With Men: Systematic Review and Synthesis of Process Evaluations
Source: J Med Internet Res. 2021 Apr 23;23(4):e22477. doi: 10.2196/22477 (PMC8105760; doi:10.2196/22477)
Supplement: Multimedia Appendix 1 [file jmir_v23i4e22477_app1.docx]

# Appendix 1. Search terms and strategy for Medline database

This appendix provides full details of the search string used for the search of the Medline database, with the date and number of references (in parentheses at the end of each row) returned.

| Database name | Medline |
| --- | --- |
| Database platform | OvidSP |
| Dates of database coverage | Ovid MEDLINE(R) and Epub Ahead of Print, In-Process & Other Non-Indexed Citations and Daily 1946 to October 22, 2018 |
| Date searched | 23/10/2018 |
| Searched by | JF |
| Number of results | 4701 |
| EndNote import order | 1 |
| Number of results once duplicates removed | 4596 |
| Search strategy notes | Search lines ending in a ‘/’ are subject heading searches. Search lines beginning ‘exp’ are exploded subject heading searches. Search lines ending in .ti,ab. search in the title and abstract only. or/*x-y* combines search sets in the range *x-y* with Boolean operator OR. * is used for truncation of words. # is used for a compulsory wildcard. ? is used for an optional wildcard. |

- - 1. Homosexuality/ (12169)
    2. Homosexuality, Male/ (13445)
    3. exp "Sexual and Gender Minorities"/ (3131)
    4. Bisexuality/ (3695)
    5. Transsexualism/ (3421)
    6. gender identity/ (17248)
    7. Health Services for Transgender Persons/ (92)
    8. exp Sex Reassignment Procedures/ (550)
    9. homosexual*.ti,ab. (13006)
    10. gay.ti,ab. (9392)
    11. "men who have sex with men".ti,ab. (9288)
    12. MSM.ti,ab. (8276)
    13. bisexual*.ti,ab. (7793)
    14. gbMSM.ti,ab. (42)
    15. (transgender* or trans-gender*).ti,ab. (3923)
    16. (transsexual* or trans-sexual*).ti,ab. (2333)
    17. (transm#n or trans-men or trans-man).ti,ab. (209)
    18. (transwom#n or trans-wom#n).ti,ab. (220)
    19. (transfemale? or trans female?).ti,ab. (19)
    20. trans people.ti,ab. (82)
    21. trans person.ti,ab. (3)
    22. tgm.ti,ab. (334)
    23. tgw.ti,ab. (180)
    24. gender identity.ti,ab. (2272)
    25. cross gender.ti,ab. (256)
    26. sex reassignment.ti,ab. (516)
    27. gender reassignment.ti,ab. (270)
    28. gender dysphoria.ti,ab. (646)
    29. gender transition.ti,ab. (89)
    30. queer.ti,ab. (905)
    31. sexual-minorit*.ti,ab. (1751)
    32. gender-minorit*.ti,ab. (304)
    33. LGBT*.ti,ab. (1350)
    34. or/1-33 [MSM] (62357)
    35. exp telemedicine/ (23614)
    36. ccbt.ti,ab. (144)
    37. (ehealth or e-health or electronic health*).ti,ab. (15158)
    38. (etherap* or e-therap* or electronic therap*).ti,ab. (426)
    39. (eportal or e-portal or electronic portal).ti,ab. (1012)
    40. telehealth*.ti,ab. (3111)
    41. telemed*.ti,ab. (9034)
    42. telemonitor*.ti,ab. (1239)
    43. telepsych*.ti,ab. (514)
    44. teletherap*.ti,ab. (1309)
    45. icbt.ti,ab. (539)
    46. (mhealth or m-health).ti,ab. (2109)
    47. or/35-46 [GENERAL E-HEALTH] (45055)
    48. cell phone/ (7494)
    49. wireless technology/ (2864)
    50. exp microcomputers/ (19620)
    51. cellphone.ti,ab. (178)
    52. computer*.ti,ab. (277170)
    53. (ipad or i-pad).ti,ab. (1036)
    54. (iphone or i-phone).ti,ab. (634)
    55. (ipod or i-pod).ti,ab. (287)
    56. mobile*.ti,ab. (84502)
    57. phone*.ti,ab. (30951)
    58. smartphone.ti,ab. (5396)
    59. technolog*.ti,ab. (394411)
    60. telephon*.ti,ab. (54456)
    61. wifi.ti,ab. (281)
    62. wireless.ti,ab. (11091)
    63. or/48-62 [HARDWARE] (817195)
    64. electronic mail/ (2459)
    65. text messaging/ (2040)
    66. exp videoconferencing/ (1572)
    67. exp internet/ (70489)
    68. mobile applications/ (3439)
    69. virtual reality/ (502)
    70. android.ti,ab. (1874)
    71. (app or apps).ti,ab. (22044)
    72. blog*.ti,ab. (1537)
    73. cyber*.ti,ab. (5586)
    74. (email* or e-mail*).ti,ab. (13513)
    75. facebook.ti,ab. (2501)
    76. instagram.ti,ab. (215)
    77. instant messag*.ti,ab. (247)
    78. internet*.ti,ab. (43734)
    79. media-based.ti,ab. (796)
    80. media-deliver*.ti,ab. (51)
    81. messag* service?.ti,ab. (1044)
    82. (multimedia or multi-media).ti,ab. (4808)
    83. new-media.ti,ab. (621)
    84. (online* or on-line*).ti,ab. (114701)
    85. podcast*.ti,ab. (618)
    86. reddit.ti,ab. (56)
    87. social network* site*.ti,ab. (944)
    88. sms.ti,ab. (4906)
    89. snapchat.ti,ab. (31)
    90. social-medi*.ti,ab. (9271)
    91. software.ti,ab. (138893)
    92. telecomm*.ti,ab. (3877)
    93. text-messag*.ti,ab. (3005)
    94. texting.ti,ab. (667)
    95. twitter.ti,ab. (2077)
    96. video-based.ti,ab. (1897)
    97. virtual*.ti,ab. (113968)
    98. vlog*.ti,ab. (29)
    99. web*.ti,ab. (125844)
    100. www.ti,ab. (1454)
    101. youtube.ti,ab. (1273)
    102. or/64-101 [SOFTWARE OR MEDIA] (565472)
    103. "Cell Phone Use"/ (56)
    104. 47 or 63 or 102 or 103 [ALL EHEALTH] (1310855)
    105. 34 and 104 [MSM AND EHEALTH] (5016)
    106. limit 105 to yr="1995 -Current" (4709)
    107. remove duplicates from 106 (4701)
