# Supplementary material for: eHealth Interventions to Address Sexual Health, Substance Use, and Mental Health Among Men Who Have Sex With Men: Systematic Review and Synthesis of Process Evaluations
Source: J Med Internet Res. 2021 Apr 23;23(4):e22477. doi: 10.2196/22477 (PMC8105760; doi:10.2196/22477)
Supplement: Multimedia Appendix 2 [file jmir_v23i4e22477_app2.docx]

# Appendix 2. Descriptions of interventions included in process synthesis

Intervention descriptions are based on information from all reports included in the overall systematic review. Reports contributing to the process synthesis are marked with an *.

| ***Intervention name, study report(s) describing this intervention and targeted outcome(s)*** | ***Location - country (region)*** | ***Target population*** | ***Providers and organisation*** | ***Intervention development*** | ***Intervention aims, components, content and activities*** | ***Intervention timing and duration*** | ***Interactive or non- interactive?*** | ***Technology*** |
| --- | --- | --- | --- | --- | --- | --- | --- | --- |
| **HealthMindr**  *Sullivan 2017^1^  Jones 2020^2^  *Outcome: SH* | United States (Atlanta, Georgia and Seattle, Washington;^1^, and Atlanta, Georgia, Jackson, Mississippi and Washington, DC)^2^ | MSM | Not stated | Separate focus groups with MSM, HIV testing counsellors and key informants to identify preferences and requirements fed into an initial version of the app, which was then theatre tested with focus group discussions. These phases fed into the beta version of the app. | This multi-feature mobile HIV prevention app included monthly risk assessment quizzes followed by tailored HIV-prevention recommendations; PrEP and nPEP resources including self-assessments; map locations and details of HIV testing locations; resources to create an HIV testing plan with reminders; ordering of free condoms and at-home HIV test kits; substance use and mental health screeners and directory; HIV treatment locator; health insurance resources; a frequently-asked questions section and a tool to submit questions to study staff. | Participants were asked to keep the app on their phones for 4 months | Interactive | Smartphone/ mobile app |
| **Keep it Up!**  *Mustanski 2013^3^  *Greene 2016^4^  Mustanski 2017^5^  Mustanski 2018^6^  *Madkins 2019^7^  *Outcomes: SH, SU* | United States: Chicago, Illinois;^3,4^ recruited from Atlanta, Georgia; Chicago, Illinois; and New York, New York; and via local and national advertising^6,7^ | Ethnically and racially diverse young MSM | Not specified. Some participants were recruited from community-based organisations providing HIV testing and counselling. | Developed in partnership with community-based organisations providing HIV testing to the LGBT community and with engagement of diverse young MSM, and informed by formative mixed-methods research. | Multi-module HIV prevention intervention for young MSM with content designed to appeal to users from all racial and ethnic groups. Online modules were based on situations and settings relevant to young MSM and used a variety of media and methods such as video, animation and games. Modules addressed, among other topics, condom use; triggers for unprotected sex; obtaining support; communication; the effects of mood, drug and alcohol abuse and sexual arousal; power dynamics in relationships; and the limits of serosorting. Users developed an HIV/STI prevention plan, and goals were suggested tailored to users’ baseline risks. In the intervention’s first iteration, a booster session revisited goals and provided tailored feedback to address obstacles and set new or reaffirm existing goals.^3^ In the second iteration, two booster sessions reinforced learning, introduced new skills and provided an opportunity to review earlier goals.^5,6^ | 7 modules completed across 3 sessions. In the first iteration, these took 2 hours and were followed by a booster session at 6 weeks.^3^ In the second iteration, modules were followed by booster sessions at 3 and 6 months, with either the initial 7 modules^5^ or the full intervention^6^ lasting 2 hours. In Mustanski 2013, Mustanski 2017, Mustanski 2018 and Madkins 2019,^3,5-7^ but not Greene 2016,^4^ modules had to be done at least 24 hours apart. | Interactive | Internet |
| **myDEx**  Bauermeister 2017^8^  *Bauermeister 2019^9^  *Outcomes: SH, MH, SU* | United States | Young adult MSM | Not stated | Socio-demographically diverse Youth Advisory Board of 3 young MSM provided input on content and delivery and trained developers on same-sex attraction and young MSM dating behaviours. | This module-based comprehensive sex education intervention aimed to improve psychological well-being and reduce HIV risk by targeting condom use; HIV/STI testing; unprotected anal sex; PrEP; and alcohol/drug use before sex. Content within each session was organised into three levels: a core message, deeper discussion of relevant topics and an activity. Content used story-telling, case scenarios, motivational interviewing strategies, graphics and videos, and it was tailored to the user via personalisation, content matching and feedback to maximise persuasiveness and relevance. Interactive activities included role-play scenarios, a diary, quizzes and opportunities to develop dating strategies. | 6 sessions, each lasting 10 minutes | Interactive | Internet |
| **Online Mindfulness-Based Cognitive Therapy**  (no name)  *Avellar 2016^10^  *Outcome: MH* | United States | Same-sex attracted men with a range of bullying experiences during grade and high school | Not stated | Modelled on an existing MBCT protocol for depression and anxiety symptoms, using that programme’s audio files and handouts and with much of the intervention content drawn from the MBCT trainers’ manual. | Module-based intervention integrating mindfulness and cognitive behavioural techniques to improve mental health. Each weekly session began with an audio introduction and an outline of the session. Sessions introduced practices and skills to alleviate unpleasant thoughts, feelings and situations. Content included lectures, activities (including meditations), exercises, handouts, weekly homework assignments and both audio and video content, and directed participants to resources in the public domain and to a mindfulness meditation by a private psychologist. | 8 weekly sessions, each lasting approximately 50-90 minutes | Interactive | Internet |
| **Queer Sex Ed**  *Mustanki 2015^11^  *Outcome: SH* | United States | LGBT youth | Not stated | Informed by mixed methods research | Multimedia, comprehensive sexual health curriculum including STI prevention. Comprised an introduction and 5 modules, moderated by a female-bodied avatar called “Ed” who introduced the programme and provided a brief overview at the start of each module. Modules used varied media formats and each ended with a quiz. Content covered understanding and accepting one’s sexual orientation and gender identity; sexuality education (e.g., pleasure, anatomy and STI risk); healthy relationships; safer sex; and sexual health improvement goal-setting. | 5 modules worked through at the user’s own pace. Users could save their work and log back in at another time to continue. Results found that the intervention took a mean of 107.8 minutes to complete. | Interactive | Internet |
| **Rainbow SPARX**  *Lucassen 2015a^12^  *Lucassen 2015b^13^  *Outcome: MH* | New Zealand (Aukland) | Sexual minority youth with depressive symptoms | Participants could complete the program at home, at a youth-led organisation promoting the study, at a selected secondary school or on a dedicated computer at the research centre where the study was based. | *Rainbow SPARX* was an adaptation for LGBT youth of the computerised CBT programme *SPARX*. Researchers and clinicians worked collaboratively with young people to develop *SPARX*, with young people’s feedback informing refinement and further improvement of prototypes. Separate consultations with sexual minority youth suggested the need for a specially adapted version for this population, which became *Rainbow SPARX*. Tailored content addressed issues and experiences especially relevant to sexual minority youth. Changes were primarily script-related (accounting for 5.9% of the overall script) and included some changes to appearance. Mini-games, characters and homework tasks were unchanged. | Computerised CBT programme to reduce depressive symptoms, designed as a multi-level game. Using interactive exercises and attractive graphics, the intervention presented the user’s avatar with challenges set in a fantasy world from which they had to eradicate gloom and negativity. Following an introduction and information about depression from a guide character, the user’s avatar entered each module and completed a mission. The guide then explained its relevance to real life, and homework tasks were set. Modules introduced CBT skills, each represented as a gem the user found and added to their ‘shield against depression.’ CBT skills introduced included: ‘relax’ (relaxation training); ‘do it’ (e.g., behavioural activation); ‘sort it’ (e.g., social skills training); ‘spot it’ (recognising or naming cognitive distortions); ‘solve it’ (problem solving); and ‘swap it’ (e.g., cognitive restructuring). | Each of 7 modules took approximately 30 minutes. Users were instructed to complete 1-2 modules per week and to complete all within 2 months. | Interactive | Computer (CD-ROM), with paper-based user notebook |
| **Smartphone Self-Monitoring**  (no name)  *Swendeman 2015^14^  *Outcomes: SH, MH, SU* | United States (Los Angeles) | People living with HIV | Did not specify a provider organisation for intervention delivery, which seemed to be the research team: Users were oriented to the app by a Research Assistant and could seek help from them for using the app | Not stated | Self-monitoring intervention to support self-management in medication adherence, mental health, substance use and sexual risk behaviours. Users completed smartphone-based self-monitoring surveys daily (alcohol, tobacco and other drug use; sexual behaviours; medication adherence) and 4 times per day (physical and mental health), with reporting on stressful events and text diary entries at any time. Customisable alarms prompted users to fill in surveys and users could access a Web-based visualisation tool to view their survey responses over time and by location, as well as to view associations between variables. | Self-monitoring daily and 4 times per day, with reporting on stressful events and text diary entries at any time. Intervention length was not specified but the last follow-up assessment specified took place at 6 weeks. | Interactive | Smartphone/ mobile app |
| **WRAPP**  (and linked interventions)  *Bowen 2007^15^  Bowen 2008^16^  *Williams 2010^17^  Schonnesson 2016^18^  *Outcome: SH* | United States (rural areas);^15-17^ Sweden^18^ | Sexually active, internet-using MSM^18^ in rural areas^15,17^ | Not stated | Content was identified from focus groups in 2001 and from a Web-based assessment conducted from Jan. 2002 – Jan. 2003. Intervention format was informed by two focus groups conducted in May 2003. The Swedish adaptation^18^ was informed by 20 in-depth interviews with Swedish MSM (HIV-positive and HIV-negative) and a presentation of the intervention to professionals at HIV prevention and treatment organisations. Information tailored to Swedish context was reviewed by an experienced HIV physician. | Online modular HIV risk reduction intervention with informational content tailored for rural MSM and presented as conversations between gay men. Dialogue was interspersed with interactive activities and graphics. The first module primarily addressed HIV prevention during sex and living with HIV, and it featured links to informational websites.  Author descriptions suggest the second module changed across iterations. It initially^15^ focused on maintaining an HIV-negative status and addressed safer sex and types and correct use of condoms.  In subsequent iterations^16-18^ this module aimed to increase motivation, and a third module targeting behavioural skills was introduced. Both allowed users to print a summary of their responses to interactive components. The ‘motivation’ module helped users identify reasons for not using condoms and ways to address these to support the user’s pursuit of their life goals. The ‘behaviour’ module addressed approaches for reducing sexual risk with partners met online or in a bar. A version adapted for Sweden^18^ used the Swedish language; was tailored to Swedish health services; and added to the ‘knowledge’ module information about STIs and the Swedish Communicable Disease Act. | Initially^15^ included two 20-minute modules completed at least 24 hours apart; users were encouraged to complete all within 7 days.  Subsequently^16-18^, three modules each contained two 20-minute sessions. Initially modules had to be completed at least 48 hours apart and results found that users took an average of 19.39 days to complete them all.^16^  Subsequently,^17^ each module had to be completed within 14 days; and later,^18^ sessions had to be completed 24-48 hours apart. | Interactive | Internet |

* = report contributed to process synthesis

CBT = cognitive-behavioural therapy

HIV = human immunodeficiency virus

LGBT = lesbian, gay, bisexual and transgender

MBCT = mindfulness-based cognitive therapy

MH = mental health

MSM = men who have sex with men

nPEP = non-occupational postexposure prophylaxis

PrEP = pre-exposure prophylaxis

SH = sexual health

STI = sexually transmitted infection

SU = substance use

## References

1. Sullivan P, Driggers R, Stekler J, et al. Usability and Acceptability of a Mobile Comprehensive HIV Prevention App for Men Who Have Sex With Men: A Pilot Study. *JMIR Mhealth Uhealth.* 2017;5(3):14.

2. Jones J, Dominguez K, Stephenson R, et al. A Theoretically Based Mobile App to Increase Pre-Exposure Prophylaxis Uptake Among Men Who Have Sex With Men: Protocol for a Randomized Controlled Trial. *JMIR Res Protoc.* 2020;9(2).

3. Mustanski B, Garofalo R, Monahan C, Gratzer B, Andrews R. Feasibility, Acceptability, and Preliminary Efficacy of an Online HIV Prevention Program for Diverse Young Men who have Sex with Men: The Keep It Up! Intervention. *AIDS Behav.* 2013;17(9):14.

4. Greene G, Madkins K, Andrews K, Dispenza J, Mustanski B. Implementation and evaluation of the Keep it Up! online HIV prevention intervention in a community-based setting. *AIDS Education and Prevention.* 2016;28(3):15.

5. Mustanski B, Madkins K, Greene G, et al. Internet-Based HIV Prevention With At-Home Sexually Transmitted Infection Testing for Young Men Having Sex With Men: Study Protocol of a Randomized Controlled Trial of Keep It Up! 2.0. *JMIR Research Protocols.* 2017;6(1):17.

6. Mustanski B, Parsons J, Sullivan P, Madkins K, Rosenberg E, Swann G. Biomedical and Behavioral Outcomes of Keep It Up!: An eHealth HIV Prevention Program RCT. *American Journal of Preventive Medicine.* 2018;55(2):8.

7. Madkins K, Moskowitz D, Moran K, Dellucci T, Mustanski B. Measuring Acceptability and Engagement of the Keep It Up! Internet-based HIV Prevention Randomized Controlled Trial for Young Men who have Sex with Men. *AIDS Educ Prev.* 2019;31(4):287-305.

8. Bauermeister J, Tingler R, Michele Demers M, Harper G. Development of a Tailored HIV Prevention Intervention for Single Young Men Who Have Sex With Men Who Meet Partners Online: Protocol for the myDEx Project. *JMIR Research Protocols.* 2017;6(7):12.

9. Bauermeister J, Tingler R, Demers M, et al. Acceptability and Preliminary Efficacy of an Online HIV Prevention Intervention for Single Young Men Who Have Sex with Men Seeking Partners Online: The myDEx Project. *AIDS and Behavior.* 2019;23(11):3064-3077.

10. Avellar T. *The feasibility and acceptability of an online mindfulness-based cognitive therapy intervention for same-sex attracted men*, University of California Santa Barbara; 2016.

11. Mustanski B, Greene G, Ryan D, Whitton S. Feasibility, Acceptability, and Initial Efficacy of an Online Sexual Health Promotion Program for LGBT Youth: The Queer Sex Ed Intervention. *The Journal of Sex Research.* 2015;42(2):11.

12. Lucassen M, Hatcher S, Fleming T, Stasiak K, Shepherd M, Merry S. A qualitative study of sexual minority young people’s experiences of computerised therapy for depression. *Australasian Psychiatry.* 2015;23(3):6.

13. Lucassen M, Merry S, Hatcher S, Frampton C. Rainbow SPARX: A Novel Approach to Addressing Depression in Sexual Minority Youth. *Cognitive and Behavioral Practice.* 2015;22(2):14.

14. Swendeman D, Ramanathan C, Baetscher L, et al. Smartphone self-monitoring to support self-management among people living with HIV: Perceived benefits and theory of change from a mixed-methods, randomized pilot study. *J Acquir Immune Defic Syndr.* 2015;69:12.

15. Bowen A, Horvath K, Williams M. A randomized control trial of Internet-delivered HIV prevention targeting rural MSM. *Health Education Research.* 2007;22(1):8.

16. Bowen A, Williams M, Daniel C, Clayton S. Internet based HIV prevention research targeting rural MSM: feasibility, acceptability, and preliminary efficacy. *J Behav Med.* 2008;31(6):15.

17. Williams M, Bowen A, Ei S. An Evaluation of the Experiences of Rural MSM Who Accessed an Online HIV/AIDS Health Promotion Intervention. *Health Promot Pract.* 2010;11(4):9.

18. Schonnesson L, Bowen A, Williams M. Project SMART: Preliminary Results From a Test of the Efficacy of a Swedish Internet-Based HIV Risk-Reduction Intervention for Men Who Have Sex With Men. *Arch Sex Behav.* 2016;45(6):11.
