# Supplementary material for: eHealth Interventions to Address Sexual Health, Substance Use, and Mental Health Among Men Who Have Sex With Men: Systematic Review and Synthesis of Process Evaluations
Source: J Med Internet Res. 2021 Apr 23;23(4):e22477. doi: 10.2196/22477 (PMC8105760; doi:10.2196/22477)
Supplement: Multimedia Appendix 3 [file jmir_v23i4e22477_app3.docx]

# Appendix 3. Characteristics and quality appraisal of process evaluations

| Study details | Characteristics of process evaluations | |
| --- | --- | --- |
| ***Programme:*** ***HealthMindr*** *(Sullivan 2017)*^1^ | | |
| Methods | Overall study design: | Cross-sectional |
|  | Research questions/ hypotheses: | Purpose was to describe and report the initial evaluation of the app for usability and acceptability; hypothesised that compared to those in Atlanta, Georgia participants in Seattle, Washington would be less interested in using the app to access services. |
|  | Timing and duration: | Recruitment May – August 2015. Participants completed the evaluation survey after having the app on their mobile phone for 4 months. |
|  | Aspects of process evaluated: | Reach, acceptability, mechanism, context |
|  | Evaluates how processes vary by intervention characteristics, providers, participants and/or contexts? | Explored how receipt varied with context and with characteristics of intervention and participants. |
|  | Data collection: | Usage data on participants’ actions within the app (button clicks, page views, and assessment and quiz responses), and web-based survey after 4-month intervention period. Survey asked about motivation to use the app; at-home test kit and condom use for those placing in-app orders; HIV testing and PrEP and nPEP use during the study period; and questions on the app’s features, usability, design, content, and functionality (using Likert scales and optional open-text fields). In-depth interviews with subsample of participants recommended to receive PrEP (about their decision of whether to start PrEP and how the app influenced their decision-making). |
|  | Data analysis: | Used usage log data to calculate descriptive statistics for the number of days using the app, pages accessed, and time spent in the app and engaged with the app; and counts and percentages of features used and app pages accessed. Calculated percentage of participants completing the follow-up survey; calculated system usability score (aggregate score ranges 1-100 based on a series of survey questions). |
| Details of participants | Location- country (region): | USA (Atlanta, Georgia and Seattle, Washington) |
|  | Target population: | MSM living in Atlanta, Georgia and Seattle, Washington metro areas |
|  | Sampling: | Web-based recruitment via Facebook advertisements targeting adult males living in Atlanta or Seattle who indicate an interest in men; and advertisements on an MSM social or sexual networking mobile app using geolocation to target users in the Atlanta or Seattle metro areas.  Eligible participants were Android phone users 18 years old or older living in the targeted areas, spoke English, were assigned male sex at birth and identified as male at screening, had sex with a man in the past year, and had never tested positive for HIV.  Invited selected participants recommended to receive PrEP for in-depth interviews, including all participants who started PrEP. |
|  | Actual sample: | - **Participants:** 121 MSM (72 in Atlanta, Georgia And 49 in Seattle, Washington) - **Follow-up:** App usage data available for 90% of sample; 81% completed 4-month evaluation survey |
|  | Sexuality: | - **Total:** 86% gay/homosexual, 11.6% bisexual - **Atlanta:** 88.9% gay/homosexual, 12.2% bisexual - **Seattle:** 81.6% gay/homosexual, 12.2% bisexual |
|  | Gender identity: | 100% male |
|  | Ethnicity: | - **Total:** 51.2% White/Caucasian, 20.7% Black or African American, 8.3% Hispanic - **Atlanta:** 47.2% White or Caucasian, 33.3% Black or African American, 4.2% Hispanic or Latino, 6.9% Asian or Pacific Islander, 8.3% Multiracial or other - **Seattle:** 57.1% White or Caucasian, 2% Black or African American, 14.3% Hispanic or Latino, 14.3% Asian or Pacific Islander, 12.2% multiracial or other |
|  | Socioeconomic status: | Not stated |
|  | Age: | - **Total:** Median age in years (interquartile range): 28 (24-34) - **Atlanta:** Median age in years (interquartile range): 28 (24-35) - **Seattle:** Median age in years (interquartile range): 28 (23-33) |
| *Quality assessment* |  |  |
| *Questions used to judge rigour and relevance* | *Reviewer judgement* | *Description* |
| Were steps taken to minimise bias and error/increase rigour in sampling? | Y | Large, purposively selected sample; used multiple methods of recruitment |
| Were steps taken to minimise bias and error/increase rigour in data collection? | Y | Used multiple forms of data including open-text fields and validated usability scale |
| Were steps taken to minimise bias and error/increase rigour in data analysis? | N | Did not describe analysis methods for qualitative data |
| Were the findings of the study grounded in/supported by data? | Y | All findings were supported by evidence |
| Was there good breadth and depth achieved in the findings? | Y | Though used primarily quantitative methods, these data provided both a breadth and depth of findings about different aspects of the app |
| The perspectives of men who have sex with men privileged? | N | Data were from MSM but appeared to be reported from closed-ended questions; briefly referenced qualitative findings but these were not clearly identified |
| *Overall reliability and usefulness of findings* | | |
| Reliability of findings | M | High rates of survey response and usage data, but qualitative analysis methods not presented and results from qualitative data collection not clearly presented |
| Usefulness of findings | H | Data provided useful findings that shed light on how design affected use, and on differences in receipt by setting |
| ***Programme:*** ***Keep it Up!*** *(Mustanski 2013)*^2^ | | |
| Methods | Overall study design: | RCT |
|  | Research questions/ hypotheses: | This pilot study aimed to determine the feasibility of the study methods (enrolment and retention) and measure the acceptability of the intervention |
|  | Timing and duration: | Evaluation took place August 2009 – September 2010. |
|  | Aspects of process evaluated: | Feasibility, reach, acceptability |
|  | Evaluates how processes vary by intervention characteristics, providers, participants and/or contexts? | Explored how receipt varied by characteristics of the intervention. |
|  | Data collection: | Assessed acceptability using a self-administered eight-item Likert scale measure as well as open-ended questions administered immediately post-intervention. Assessed feasibility using enrolment and retention data, and assessed reach by calculating the proportion of participants completing all intervention modules. |
|  | Data analysis: | Descriptive statistics of quantitative acceptability measure, and counts and percentages to assess enrolment and retention. Responses to qualitative questions on acceptability were coded based on the main categories of format, content and take-away. Responses were double-coded and reliability assessed using Cohen’s kappa. |
| Details of participants | Location- country (region): | United States (Chicago, Illinois) |
|  | Target population: | Ethnically and racially diverse young MSM who have received an HIV-negative test result at a clinic. |
|  | Sampling: | Young MSM ages 18-24 receiving an HIV-negative test result from a participating clinic were eligible to take part if their male birth sex and gender identity were male and they had had sex with a male in the prior three months, had at least one act of unprotected anal sex in the prior three months, were not currently in an exclusive/monogamous relationship lasting longer than 12 months, were able to read at an 8^th^ grade level and accessed the internet at least several times in the past month. |
|  | Actual sample: | 102 participants completed the baseline assessment and were randomised, of which 50 were randomised to the intervention arm. Of those in the intervention arm, 48 (96.1%) completed post-intervention follow-up when process evaluation questions were asked. |
|  | Sexuality: | **Baseline characteristics among intervention sample:**  78.0% gay/homosexual, 22.0% bisexual/other |
|  | Gender identity: | Not stated; eligible participants were male at birth and had a male gender identity |
|  | Ethnicity: | **Baseline characteristics among intervention sample:**  46.0% White-Latino, 24.0% White-non-Latino, 14.0% African American, 16.0% Other |
|  | Socioeconomic status: | **Baseline characteristics among intervention sample:**   - Employment: 56.0% employed - Education: 24.0% some high school or graduate, 76.0% some college or graduate |
|  | Age: | **Baseline characteristics among intervention sample:**  Mean age (SD): 21.62 years (1.97) |
| *Quality assessment* |  |  |
| *Questions used to judge rigour and relevance* | *Reviewer judgement* | *Description* |
| Were steps taken to minimise bias and error/increase rigour in sampling? | Y | Sample was all those involved in RCT which had clear inclusion criteria and used multiple methods of recruitment |
| Were steps taken to minimise bias and error/increase rigour in data collection? | Y | Assessed acceptability using items from existing scale; open-ended question also used |
| Were steps taken to minimise bias and error/increase rigour in data analysis? | Y | Qualitative data were double-coded and reliability was assessed |
| Were the findings of the study grounded in/supported by data? | N | Did not provide supporting quotations for all themes |
| Was there good breadth and depth achieved in the findings? | N | Quantitative findings were reported as one combined rating; qualitative findings reported thinly on aspects of the intervention participants did and did not like |
| The perspectives of men who have sex with men privileged? | Y | Data came from MSM and were analysed and reported in detail; data collection included open-ended questions |
| *Overall reliability and usefulness of findings* | | |
| Reliability of findings | H | High response rate among intervention participants, and well-described methods; findings were likely valid |
| Usefulness of findings | M | Findings were relatively thin, but addressed how acceptability varied by characteristics of the intervention |
| ***Programme:*** ***Keep it Up!*** *(Greene 2016)*^3^ | | |
| Methods | Overall study design: | Uncontrolled before/after |
|  | Research questions/ hypotheses: | Aimed to describe the adaptation and implementation procedures for intervention delivery in a non-profit, community-based organisation and to assess intervention acceptability among participants. |
|  | Timing and duration: | Intervention was delivered from 2012-2013 |
|  | Aspects of process evaluated: | Reach, acceptability, context |
|  | Evaluates how processes vary by intervention characteristics, providers, participants and/or contexts? | Explored how receipt varied by characteristics of the intervention and of participants. |
|  | Data collection: | Online evaluation surveys at baseline, post-intervention and 6- and 12-week follow-up. Data for process evaluation synthesis come from responses to three open-ended questions administered in 6- and 12-week follow-ups. |
|  | Data analysis: | Content analysis. Following coding by two independent raters, reliability was assessed using Cohen’s kappa. Excerpts were organised by theme and coders identified examples of typical responses. |
| Details of participants | Location- country (region): | United States |
|  | Target population: | Racially and ethnically diverse young MSM |
|  | Sampling: | Participants were recruited by counsellors in an HIV testing clinic, at the organisation’s in-house events, via print and online ads, and via friend referrals. Eligible participants were aged 18-24 years, assigned male sex at birth, had a valid email address and either received an HIV-negative test result from clinic staff or self-reported having an HIV-negative test result in the past 3 months. All were invited to take part in evaluation surveys containing the questions used in this review’s process evaluation synthesis. |
|  | Actual sample: | 343 participants enrolled in and completed the intervention (this is 45.4% of those eligible; no information is provided on then number completing the baseline survey but not the intervention, if any). Of these, 219 (63.8%) completed 6-week follow-up survey and 200 (58.31%) completed 12-week follow-up survey. |
|  | Sexuality: | - **Baseline:** 73.4% gay/homosexual, 26.3% bisexual/other - **12-month follow-up:** 84.5% gay/homosexual, 15.5% bisexual/other |
|  | Gender identity: | - **Baseline:** 92.8% male, 1.8% female, 5.0% transgender - **12-month follow-up:** 93.0% male, 1.5% female, 5.5% transgender |
|  | Ethnicity: | - **Baseline:** 31.6% African American, 21.6% Latino, 33.9% White, 12.9% other - **12-month follow-up:** 21.0% African American, 19.0% Latino, 48.5% White, 11.5% Other |
|  | Socioeconomic status: | **Baseline:**   - Employment: 50.6% employed - Education: 33.6% some high school or graduate, 66.4% some college or graduate   **12-month follow-up:**   - Employment: 60.0% employed - Education: 20.5% some high school or graduate, 79.5% some college or graduate |
|  | Age: | - **Baseline:** Mean (SD) age 21.52 years (1.94) - **12-month follow-up:** Mean (SD) age 21.73 years (1.91) |
| *Quality assessment* | | |
| *Questions used to judge rigour and relevance* | *Reviewer judgement* | *Description* |
| Were steps taken to minimise bias and error/increase rigour in sampling? | Y | Questions directed at all intervention recipients, with good response rate |
| Were steps taken to minimise bias and error/increase rigour in data collection? | Y | Used existing survey measures of acceptability augmented by open-ended questions |
| Were steps taken to minimise bias and error/increase rigour in data analysis? | Y | Qualitative data double-coded with high reliability |
| Were the findings of the study grounded in/supported by data? | Y | Themes were defined and supporting questions provided |
| Was there good breadth and depth achieved in the findings? | N | Qualitative findings thinly described likes and dislikes and quantitative findings reported as one combined rating |
| The perspectives of men who have sex with men privileged? | Y | Sampled MSM and survey included a few open-ended response questions |
| *Overall reliability and usefulness of findings* | | |
| Reliability of findings | H | Data were not in-depth but were likely valid |
| Usefulness of findings | H | Findings addressed how acceptability varied by aspects of the intervention |
| ***Programme: Keep it Up!*** *(Madkins 2019)^4^* | | |
| Methods | Overall study design: | RCT |
|  | Research questions/ hypotheses: | Examined acceptability of and engagement with *Keep it Up!* intervention and aimed to explore differences in acceptability and engagement by age, race/ethnicity and education |
|  | Timing and duration: | Recruitment took place May 2013 – December 2013. Following baseline assessment, participants were enrolled and randomised. Post-test immediately followed intervention modules. |
|  | Aspects of process evaluated: | Acceptability |
|  | Evaluates how processes vary by intervention characteristics, providers, participants and/or contexts? | Explored how receipt varied by characteristics of the intervention and participants |
|  | Data collection: | Baseline and post-test survey data from intervention arm participants, data captured from intervention use on time spent in the intervention and participants ratings of each module, provided before proceeding to the next module. Intervention acceptability and tolerability assessed using adapted version of existing scale. |
|  | Data analysis: | Conducted confirmatory factor analysis followed by ANOVA to compare differences in intervention acceptability and tolerability scale, and ANOVA to compare differences in time to complete intervention. Logistic regression was used to assess star ratings, adjusting for demographic factors and intervention site; also assessed race by education interactions and included these where significant. Two independent raters conducted content analysis using qualitative data to identify themes related to acceptability, assessing reliability via Cohen’s kappa. Subtracted number of “dislike” codes from number of “like” codes to create score for overall favourability, comparing mean scores by race and education. |
| Details of participants | Location- country (region): | United States |
|  | Target population: | Ethnically and racially diverse young MSM |
|  | Sampling: | Participants were recruited from HIV testing sites, local health department clinics, street outreach, local and national advertising and research participant registries. Eligible participants were cisgender MSM reporting sexual risk, aged 18-20 years, receiving an HIV-negative test from a study site or via remote HIV self-testing. |
|  | Actual sample: | 445 intervention participants at baseline (84% response rate) and 375 (84% of baseline sample) at follow-up. |
|  | Sexuality: | 87% gay/homosexual, 14% bisexual/other |
|  | Gender identity: | Not stated, but eligible participants were cisgender MSM |
|  | Ethnicity: | 37% White, 24% Black, 30% Latino, 9% Other Race |
|  | Socioeconomic status: | **Education:** 16% high school or less, 26% some college, 46% college education, 13% graduate degree |
|  | Age: | Mean age 24.33 years (SD=3.00) |
| Details of intervention | Description: | See Mustanski 2013 |
|  | Technology: | Internet |
|  | Timing and duration: | 7 modules had to be done at least 24 hours apart and took 2 hours to complete. These were followed by booster sessions at 3 and 6 months. |
|  | Target population: | Ethnically and racially diverse young MSM |
|  | Theoretical framework: | See Mustanski 2013 |
|  | Development: | See Mustanski 2013 |
|  | Provider organisation: | See Mustanski 2013 |
|  | Content and activities: | Online modules were based on situations and settings relevant to young MSM and used a variety of media and methods such as video, animation and games. Modules addressed, among other topics, condom use; triggers for unprotected sex; obtaining support; communication; the effects of mood, drug and alcohol abuse and sexual arousal; power dynamics in relationships; and the limits of serosorting. Users developed an HIV/STI prevention plan, and goals were suggested tailored to users’ baseline risks. 2 booster sessions reinforced learning, introduced new skills and provided an opportunity to review earlier goals. |
|  | Control: | Online content similar to available didactic HIV prevention materials. Control was matched to the intervention in the number of modules and the requirement to participate in them over three sessions. At 3- and 6-month follow-up sessions (i.e., the same timing as intervention booster sessions) materials were reviewed again and information was provided on biomedical strategies. |
| *Quality assessment* |  |  |
| *Questions used to judge rigour and relevance* | *Reviewer judgement* | *Description* |
| Were steps taken to minimise bias and error/increase rigour in sampling? | Y | Recruited nationwide using a range of avenues with defined recruitment criteria |
| Were steps taken to minimise bias and error/increase rigour in data collection? | Y | Scale based on existing measure and conducted confirmatory factor analysis in this sample |
| Were steps taken to minimise bias and error/increase rigour in data analysis? | Y | Two coders analysed qualitative data; controlled for potential confounders and explored interaction in quantitative analysis |
| Were the findings of the study grounded in/supported by data? | Y | Quantitative findings in table match article narrative |
| Was there good breadth and depth achieved in the findings? | Y | Used both closed- and open-ended questions and explored a range of aspects of intervention feedback |
| The perspectives of men who have sex with men privileged? | Y | Collected both quantitative and qualitative data from MSM participants |
| *Overall reliability and usefulness of findings* | | |
| Reliability of findings | H | Used rigour in sampling, analysis and reporting |
| Usefulness of findings | H | Conducted an extensive analysis of differences in acceptability by race, education level and age |
| ***Programme: myDEx*** *(Bauermeister 2019)* | | |
| Methods | Overall study design: | RCT |
|  | Research questions/ hypotheses: | Aimed to assess proposed intervention mechanisms of change associated with risk of HIV |
|  | Timing and duration: | Baseline questionnaire was followed by randomisation. Follow-up assessments took place at 30, 60 and 90 days post-randomisation. |
|  | Aspects of process evaluated: | Feasibility, acceptability and mechanism |
|  | Evaluates how processes vary by intervention characteristics, providers, participants and/or contexts? | Explored how receipt varied by characteristics of the intervention |
|  | Data collection: | Assessed acceptability, usability and utility quantitatively at 30-day follow-up assessment. Questions assessed overall satisfaction, willingness to recommend the intervention, likelihood to continue using the intervention, usability, ease of navigation and technical responsiveness. |
|  | Data analysis: | Compared scores between intervention and control arms |
| Details of participants | Location- country (region): | United States |
|  | Target population: | Single, young gay, bisexual and other men who have sex with men who are presumed HIV-negative and report condomless anal intercourse with partners met online |
|  | Sampling: | Recruited from across the United States via online ads on social and sexual networking sites. Eligible participants were single, cisgender males aged 18-24 years who reported online dating app use and sexual risk and HIV-negative or HIV-unknown status. |
|  | Actual sample: | 180 participants enrolled and were randomised. 91.1% completed at least one follow-up assessment, with 79.4% completing 30-day follow-up. Due to a programming error 25 control participants were exposed to the intervention and excluded, leaving 155 participants included in final analysis overall. |
|  | Sexuality: | **Full sample**  88.3% gay, 7.8% bisexual, 3.9% queer  **N=155 included in analysis**  89.0% gay, 7.1% bisexual, 3.9% queer |
|  | Gender identity: | Not stated; eligible participants were cisgender MSM |
|  | Ethnicity: | **Full sample**  Race: 67.2% White, 16.1% Multiracial, 10.0% Black, 5.6% Asian, 0.6% Middle Eastern, 0.6% Native American  Ethnicity: 30% Hispanic/Latino  **N=155 included in analysis**  Race: 67.1% White, 16.1% Multiracial, 10.0% Black, 5.2% Asian, 0.6% Middle Eastern, 0.6% Native American  Ethnicity: 30% Hispanic/Latino |
|  | Socioeconomic status: | **Full sample**  2.8% some high school, 10.6% high school diploma or GED equivalent, 7.8% technical or associate degree, 40.0% some college, 29.4% graduated college, 9.5% reported attending graduate school |
|  | Age: | **Full sample**  Mean 21.67 years (SD=1.81)  **N=155 included in analysis**  Mean 21.5 (SD=1.82) |
| Details of intervention | Description: | Online, module-based comprehensive sex education intervention to improve psychological well-being and HIV risk |
|  | Technology: | Internet |
|  | Timing and duration: | 6 sessions, each lasting 10 minutes |
|  | Target population: | Young adult MSM |
|  | Theoretical framework: | This intervention aimed to improve psychological well-being and reduce HIV risk via behaviour change, increasing PrEP use and decreasing alcohol and drug use before sex by targeting cognitive and affective motivations. It was informed by the notion that decision-making is shaped by both cognitive and affective motivations and that when these are less aligned, there is less of a correspondence between intentions and behaviour.  Content targeting cognitive motivations focused on attitudes, norms and perceived behavioural control to engage in risk reduction behaviours. Attitudes and norms were theorised to each influence each other, and all three constructs were theorised to influence behavioural intentions. Content targeting affective motivations addressed relationship ideation, anticipated regret, limerence and decisional balance to forego condoms. Affective motivations were theorised to influence behavioural intentions which were theorised to directly influence HIV risk reduction behaviours.  Psychological risk correlates, psychological distress and substance use and abuse were theorised to influence regulation of affective motivations and therefore behavioural control, affecting risk behaviours. Type of sexual partner was theorised to affect perceived behavioural control and the relationship between behavioural intentions and actual behaviours. |
|  | Development: | Socio-demographically diverse Youth Advisory Board of 3 young MSM provided input on content and delivery and trained developers on same-sex attraction and young MSM dating behaviours. |
|  | Provider organisation: | Not stated |
|  | Content and activities: | This module-based comprehensive sex education intervention aimed to improve psychological well-being and reduce HIV risk by targeting condom use; HIV/STI testing; unprotected anal sex; PrEP; and alcohol/drug use before sex. Each session included activities and videos, and content within each session was organised into three levels: a core message, deeper discussion of relevant topics and an activity. Content used story-telling, case scenarios, motivational interviewing, graphics and videos, and it was tailored via personalisation, content matching and feedback to maximise persuasiveness and relevance. Interactive activities included role-play scenarios, a diary, quizzes and opportunities to develop dating strategies. |
|  | Control: | Information-only attention control: 6 sessions, matching the intervention’s design, with content mirroring that from the United States Centers for Disease Control and Prevention’s HIV Risk Reduction Tool. |
| *Quality assessment* | | |
| *Questions used to judge rigour and relevance* | *Reviewer judgement* | *Description* |
| Were steps taken to minimise bias and error/increase rigour in sampling? | Y | Participants recruited from across the country using defined recruitment criteria and multiple online sites |
| Were steps taken to minimise bias and error/increase rigour in data collection? | Y | Provided incentives to support retention throughout follow-up period |
| Were steps taken to minimise bias and error/increase rigour in data analysis? | Y | Assessed and found no sociodemographic differences between arms |
| Were the findings of the study grounded in/supported by data? | Y | Quantitative results are presented clearly in a table |
| Was there good breadth and depth achieved in the findings? | N | All findings based on closed-ended Likert scale items |
| The perspectives of men who have sex with men privileged? | N | Though participants were MSM, all findings were based on closed-ended Likert scale items |
| *Overall reliability and usefulness of findings* | | |
| Reliability of findings | H | Participants recruited from across the country |
| Usefulness of findings | L | Provides some information on usability but does not explore which aspects were most useful or could be improved |
| ***Programme:*** ***Online Mindfulness-Based Cognitive Therapy*** *(no name) (Avellar 2016)*^5^ | | |
| Methods | Overall study design: | RCT |
|  | Research questions/ hypotheses: | Aimed to assess feasibility, acceptability and factors leading to high attrition in efficacy study.  Research questions:   - What would be needed to recruit and retain an adequate sample of participants to conduct an efficacy study of a multi-session, online mindfulness training?   - How likely are same-sex attracted men on Amazon Mechanical Turk (MTurk) to participate in multi-session online mindfulness training?   - What number of participant characteristics, recruitment processes, and characteristics of the intervention would yield enough participants to have sufficient statistical power? - How valuable do same-sex attracted men find multi-session online mindfulness training?   - To what extent did they perceive mindfulness as something that will benefit their mental health?   - To what extent do they perceive mindfulness training as worth the effort to commit to the mindfulness practices? |
|  | Timing and duration: | Not specified. Pre-test preceded allocation to intervention or waitlist control group; sessions were weekly; and following the intervention participants were invited to participate in the feasibility and acceptability study. |
|  | Aspects of process evaluated: | Feasibility, reach and acceptability |
|  | Evaluates how processes vary by intervention characteristics, providers, participants and/or contexts? | Explored how receipt varied by characteristics of the intervention and of participants. |
|  | Data collection: | Online baseline and post-intervention surveys, with the latter including quantitative and qualitative items assessing acceptability and feasibility. Quantitative measures assessed ease of use, overall utility, appropriateness for target group, and acquisition of new knowledge. Qualitative questions asked participants about aspects of the efficacy study liked most and least. |
|  | Data analysis: | - **Quantitative data**: Analysed by item-by-item descriptive statistics, multinomial logistic regression, and chi-square tests of independence - **Qualitative data**: Content analysis, generating descriptive codes to describe each unit of data, then category codes to describe groups of descriptive codes. Codes were audited at two stages by another researcher. |
| Details of participants | Location- country (region): | United States |
|  | Target population: | Same-sex attracted men with a range of bullying experiences during grade and high school |
|  | Sampling: | Eligible participants for the overall efficacy study were aged 18 years or older, identified gay- or same-sex attracted males, were fluent in English and were not currently engaged in psychotherapy. They were recruited through MTurk using MTurk’s keyword function. Intervention group participants were eligible to take part in the process evaluation component if they completed at least the pre-test. |
|  | Actual sample: | 80 intervention group participants completed at least the pre-test and of these 41 (51.3%) completed post-intervention feasibility and acceptability survey. |
|  | Sexuality: | Not stated |
|  | Gender identity: | Not stated |
|  | Ethnicity: | - **Baseline:** 67.5% European American/White, 12.5% Latino/a or Hispanic, 2.5% African American or Black, 1.25% American Indian, 5% Asian American, 1.25% Middle Eastern, 3.75% Multi-Ethnic - **Follow-up:** 68.3% European American/White, 9.7% Latino/a or Hispanic, 2.5% African American or Black, 2.5% American Indian, 7.3% Asian American, 2.5% Middle Eastern, 7.3% Multi-Ethnic |
|  | Socioeconomic status: | **Baseline:**   - SES – level out of 7, with 1 as worst off and 7 as best off: 1 – worst off (2.5%); 2 (23.75%); 3 (23.75%); 4 (23.75); 5 (23.75%); 6 (5%); 7 – best off (0%) - Education: 1.25% less than high school, 8.75% high school or GED, 1.25% trade or vocational, 27.5% some college – no degree, 16.2% associates degree, 31.2% bachelors degree, 15% graduate or professional degree   **Follow-up:**   - SES – level out of 7, with 1 as worst off and 7 as best off: 1 – worst off (2.5%); 2 (7.3%); 3 (17.1%); 4 (19.5%); 5 (21.9%); 6 (7.3%); 7 – best off (0%) - Education: 2.5% less than high school, 7.3% high school or GED, 0% trade or vocational, 19.5% some college – no degree, 24.4% associates degree, 29.3% bachelors degree, 17.1% graduate or professional degree |
|  | Age: | **Baseline among those…**   - completing pre-test only: mean=28.14 years, SD=8.63, median=25.5 - completing between 1 and 3 sessions: mean=28.14 years, SD=8.05, median=25.5 - completing between 4 and 8 sessions: mean=28.87, SD=4.03, median=27   **Follow-up among those…**   - completing pre-test only: mean=30.15, SD=6.41, median=28 - completing between 1 and 3 sessions: mean=30.08, SD=10.4, median=25 - completing between 4 and 8 sessions, m=26.93, SD=4.06, median=27 |
| *Quality assessment* |  |  |
| *Questions used to judge rigour and relevance* | *Reviewer judgement* | *Description* |
| Were steps taken to minimise bias and error/increase rigour in sampling? | N | Low response rate, resulting in small sample likely to differ from trial |
| Were steps taken to minimise bias and error/increase rigour in data collection? | Y | Used quantitative and qualitative survey questions; items based on existing, reliable measures |
| Were steps taken to minimise bias and error/increase rigour in data analysis? | Y | Iterative checking of emerging analysis by second researcher |
| Were the findings of the study grounded in/supported by data? | Y | Detailed results presented, including clear summaries of participant accounts |
| Was there good breadth and depth achieved in the findings? | Y | Results included specific aspects of intervention that participants liked/disliked, and why |
| The perspectives of men who have sex with men privileged? | Y | Quantitative and quantitative data from MSM were reported in detail |
| *Overall reliability and usefulness of findings* | | |
| Reliability of findings | M | Used multiple methods of data collection, but response rates were low |
| Usefulness of findings | H | Reported data on aspects of intervention affecting receipt |
| ***Programme:*** ***Queer Sex Ed*** *(Mustanski 2015)*^6^ | | |
| Methods | Overall study design: | Uncontrolled before/after study |
|  | Research questions/ hypotheses: | The study aimed to:   1. Determine the feasibility of recruiting and enrolling LGBT youth in same-sex relationships into an online sexual health intervention 2. Use mixed methods to evaluate the acceptability of and engagement with the intervention |
|  | Timing and duration: | Enrolment from November 2012-April 2013. Participants completed pretest survey then accessed the intervention. Posttest surveys were completed at least 2 weeks after intervention completion, but it is not clear whether these included the process evaluation questions or only impact-related measures. Content ratings seemed to have been asked throughout the intervention, and qualitative feedback was requested after the intervention (timing was not specified). |
|  | Aspects of process evaluated: | Reach and acceptability |
|  | Evaluates how processes vary by intervention characteristics, providers, participants and/or contexts? | Explored how receipt varied by characteristics of the intervention |
|  | Data collection: | Participants rated each page on whether content was helpful, informational and interesting on a scale from 1-5 stars. Questions seemed to be embedded in the intervention pages rather than asked post-intervention but this is not clear. After intervention completion participants were asked open-ended questions about what they liked and disliked about the intervention; timing and method of this data collection were unclear. |
|  | Data analysis: | Overall content ratings calculated by taking the mean of all scale ratings for individual pages. Qualitative responses on intervention acceptability were coded by two independent coders according to the categories of format, content and takeaway messages; reliability was assessed using Cohen’s kappa. List of excerpts for each theme were generated and coders selected examples of typical responses. |
| Details of participants | Location- country (region): | United States   - **Male-born participants (N=107):** 79.4% urban, 20.6% rural - **Female-born participants (N=95):** 85.3% urban, 12.6% rural, 2.1% missing |
|  | Target population: | LGBT youth |
|  | Sampling: | Eligible participants identified as LGBT or queer, or reported same-sex attraction or behaviours; were aged 16-20 years; lived in the United States; and currently engaged in a romantic relationship with a partner of the same biological sex. Targeted recruitment proceeded via social media. |
|  | Actual sample: | 276 participants consented and completed the pre-test, of whom 210 (76.1%) completed the intervention. Of these 210, 202 (73.2%) completed the posttest and comprise the sample for the process evaluation. |
|  | Sexuality: | - **Male-born participants (N=107):** 84.1% gay/lesbian, 9.3% bisexual, 5.6% queer, 0.9% unsure/questioning - **Female-born participants (N=95):** 54.7% gay/lesbian, 22.1% bisexual, 22.1% queer, 1.1% unsure/questioning |
|  | Gender identity: | - **Male-born participants (N=107):** 96.3% male, 3.7% transgender - **Female-born participants (N=95):** 88.4% female, 10.5% transgender |
|  | Ethnicity: | - **Male-born participants (N=107):** 76.6% White 15% Latino/a, 0.9% Black, 7.5% Other - **Female-born participants (N=95):** 82.1% White, 5.3% Latino/a, 5.3% Black, 7.4% Other |
|  | Socioeconomic status: | - **Male-born participants (N=107):** 41.4% less than high school, 22.4% high school graduate, 35.5% higher than high school - **Female-born participants (N=95):** 40% less than high school, 20.4% high school graduate, 37.9% higher than high school |
|  | Age: | - **Male-born participants (N=107):** Age in years: 16 (18.7%), 17 (25.2%), 18 (20.6%), 19 (16.8%), 20 (18.7%) - **Female-born participants (N=95):** Age in years: 16 (22.1%), 17 (22.1%), 18 (17.9%), 19 (20%), 20 (17.9%) - **Participants completing pretest only:** Mean age 18.3 years - **Participants completing posttest:** Mean age 18.9 years |
| *Quality assessment* |  |  |
| *Questions used to judge rigour and relevance* | *Reviewer judgement* | *Description* |
| Were steps taken to minimise bias and error/increase rigour in sampling? | Y | Sample was all those receiving intervention which was guided by clear inclusion criteria and used multiple recruitment methods |
| Were steps taken to minimise bias and error/increase rigour in data collection? | Y | Collected both quantitative and qualitative data including content ratings for each page of the intervention |
| Were steps taken to minimise bias and error/increase rigour in data analysis? | Y | Qualitative data were coded by two independent coders who achieved high reliability |
| Were the findings of the study grounded in/supported by data? | Y | Findings were presented in text which gives areas of likes and dislikes and in table which provided supporting quotes |
| Was there good breadth and depth achieved in the findings? | N | Findings were presented as list of likes and dislikes with no in-depth analysis; quotations were very short and not interpreted |
| The perspectives of men who have sex with men privileged? | Y | Open-ended questions allowed some space for MSM participants to set out their own views |
| *Overall reliability and usefulness of findings* | | |
| Reliability of findings | M | High response rate and well-described analysis methods, but data collected were thin and presented with little interpretation |
| Usefulness of findings | L | Presented a list of likes and dislikes with little analysis of how aspects of the intervention accepted its receipt |
| ***Programme:*** ***Rainbow SPARX*** *(Lucassen 2015a)^7^* | | |
| Methods | Overall study design: | Uncontrolled before/after study |
|  | Research questions/ hypotheses: | Objectives of the process evaluation were:   1. To ascertain the acceptability of the intervention 2. To assess feasibility (based on recruitment and uptake rates) |
|  | Timing and duration: | The overall (including effectiveness) study included assessments at pre-intervention, post-intervention and 3-month follow-up. Acceptability was assessed at post-intervention. |
|  | Aspects of process evaluated: | Feasibility, reach and acceptability |
|  | Evaluates how processes vary by intervention characteristics, providers, participants and/or contexts? | Explored how receipt varied by characteristics of the intervention and of participants |
|  | Data collection: | Post-intervention questionnaire assessed acceptability via Likert-format questions on intervention appeal, usefulness and likability. It also asked about time required to complete each module, whether participants would recommend the intervention to their friends, whether they thought it would appeal to other young people, and how many modules they completed. |
|  | Data analysis: | Feasibility was determined by the number of participants expressing interest in participating in the study and the number who enrolled. Surveys were analysed quantitatively to assess acceptability. |
| Details of participants | Location- country (region): | New Zealand (Aukland) |
|  | Target population: | Sexual minority youth with depressive symptoms |
|  | Sampling: | Eligible participants were sexual minority youth (adolescents who are sexually attracted to the same sex, both sexes, or who were questioning their sexuality) aged 13-19 years with depressive symptoms (Child Depression Rating Scale-Revised raw score >30), living in Aukland, New Zealand. A youth-led organisation for sexual minority youth promoted the study and four secondary schools supportive of the initiative encouraged participation. Sexual minority media (type not specified) advertised and endorsed the study. |
|  | Actual sample: | 21 participants enrolled in the study. Of these, 19 (91%) completed the intervention and post-intervention assessment. |
|  | Sexuality: | All were sexual minority participants, defined by the authors as those sexually attracted to the same or both sexes or are questioning their sexuality |
|  | Gender identity: | **Participants enrolled in the study**  52.4% identified as male |
|  | Ethnicity: | **Participants enrolled in the study**  71.4% New Zealand European, 9.5% Māori, 4.8% of a Pacific ethnicity, 14.3% Asian |
|  | Socioeconomic status: | Not stated |
|  | Age: | **Participants enrolled in the study**  Ages 13-19 with a mean (SD) age of 16.5 (1.6) years |
| *Quality assessment* |  |  |
| *Questions used to judge rigour and relevance* | *Reviewer judgement* | *Description* |
| Were steps taken to minimise bias and error/increase rigour in sampling? | Y | Sampled all those involved in pilot study which had clear inclusion criteria and multi-method recruitment |
| Were steps taken to minimise bias and error/increase rigour in data collection? | N | Used all fixed-response questions; did not discuss piloting or describe which aspects and components of the intervention were asked about |
| Were steps taken to minimise bias and error/increase rigour in data analysis? | N | Did not describe analysis methods |
| Were the findings of the study grounded in/supported by data? | Y | Findings on acceptability were presented transparently |
| Was there good breadth and depth achieved in the findings? | N | Limited to reach and to thin findings on acceptability based on fixed-response questions |
| The perspectives of men who have sex with men privileged? | N | Sampled MSM but only findings on fixed-response questions were presented |
| *Overall reliability and usefulness of findings* | | |
| Reliability of findings | H | Sample was representative of those in the trial; data collected were thin but likely to be valid |
| Usefulness of findings | M | Reported data on aspects of intervention affecting receipt, but findings were thin |
| ***Programme:*** ***Rainbow SPARX*** *(Lucassen 2015b)^8^* | | |
| Methods | Overall study design: | Uncontrolled before/after study |
|  | Research questions/ hypotheses: | The study sought to determine participants’ views on:   1. What they liked and did not like about the intervention 2. How the intervention might benefit others 3. What they thought of the sexuality (or ‘Rainbow’) specific content 4. What they thought about completing the homework tasks 5. Whether or not the programme helped them feel better or less depressed |
|  | Timing and duration: | Data for this component of the study were collected immediately after the post-intervention assessment point |
|  | Aspects of process evaluated: | Acceptability |
|  | Evaluates how processes vary by intervention characteristics, providers, participants and/or contexts? | Explored how receipt varied by characteristics of the intervention and of participants |
|  | Data collection: | Semi-structured interviews which were audio-recorded and professionally transcribed |
|  | Data analysis: | Used the ‘general inductive approach’ which focused on gathering participants’ views on pre-existing questions or topics. Analysis aimed to investigate common themes and points of agreement/disagreement. Transcripts were read and re-read, with lower order units of meaning clustered with similar units. Researchers searched for contradictory views and subtopics, and reviewed clusters to identify the meaning of each category. An accuracy check identified only minor discrepancies, resolved through discussion, and data were coded using the identified themes. |
| Details of participants | Location- country (region): | New Zealand (Aukland) |
|  | Target population: | Sexual minority youth |
|  | Sampling: | Participants for the overall study of which this process evaluation was a part were recruited from four secondary schools, from a youth-led organisation for sexual minority youth, and via sexual minority media advertisements about the study |
|  | Actual sample: | 25 participants took part in an interview (this was everyone who was invited to take part in one) |
|  | Sexuality: | All were sexual minority participants, defined by the authors as those sexually attracted to the same or both sexes or are questioning their sexuality |
|  | Gender identity: | 12 (48%) identified as male, 13 (52%) identified as female (including two transgender girls). In total, 14 (56%) identified male or as transgender girls. |
|  | Ethnicity: | 15 (60%) New Zealand European, 3 (12%) Māori, 2 (8%) Asian, 1 (4%) Pacific, 4 (16%) an ‘other’ ethnicity |
|  | Socioeconomic status: | Not stated |
|  | Age: | Ages 13-19 with a mean age of 16.36 years |
| *Quality assessment* |  |  |
| *Questions used to judge rigour and relevance* | *Reviewer judgement* | *Description* |
| Were steps taken to minimise bias and error/increase rigour in sampling? | Y | Sampled all those who were involved in the pilot study, which had clear inclusion criteria and used multiple recruitment methods |
| Were steps taken to minimise bias and error/increase rigour in data collection? | Y | Provided detail on procedures and on topics explored |
| Were steps taken to minimise bias and error/increase rigour in data analysis? | Y | Used inductive systematic approach with 10% dual-coded; participants reviewed preliminary summary of findings |
| Were the findings of the study grounded in/supported by data? | Y | Themes described and supporting quotations provided |
| Was there good breadth and depth achieved in the findings? | N | Descriptions of themes were brief and not explored in depth; quotes were short and not interpreted |
| The perspectives of men who have sex with men privileged? | Y | Data came from open-ended questions asked of sexual minority youth |
| *Overall reliability and usefulness of findings* | | |
| Reliability of findings | H | Sample was representative of those in the trial; reported contrasting views |
| Usefulness of findings | M | Reported findings on how aspects of the intervention and of participants affected intervention receipt, but these findings were thin |
| ***Programme:*** ***Smartphone Self-Monitoring*** *(no name) (Swendeman 2015)^9^* | | |
| Methods | Overall study design: | RCT |
|  | Research questions/ hypotheses: | Process evaluation aimed to explore barriers and challenges encountered with the intervention to inform future work on self-monitoring |
|  | Timing and duration: | Recruitment took place over a 9-month period and process evaluation data were collected at end of weeks 2, 4 and 6 |
|  | Aspects of process evaluated: | Acceptability/satisfaction and mechanisms of action |
|  | Evaluates how processes vary by intervention characteristics, providers, participants and/or contexts? | Explored how receipt varied by characteristics of participants and of the intervention |
|  | Data collection: | Qualitative semi-structured interviews conducted by telephone at weeks 2 and 4 post-baseline and conducted in-person at 6 weeks post-baseline. Web-based surveys also used yes/no and open-ended questions to assess goals and supports in each of the 4 targeted outcome domains (medication adherence, mental health, alcohol/tobacco/other drug use and sexual risk behaviours). |
|  | Data analysis: | Coding of interview responses used an iterative, ‘grounded’ (by which the authors seem to suggest inductive) approach, identifying key themes and subthemes. The lead researcher and one other researcher generated primary codes and two other researchers then coded the data independently. The lead author reviewed and clarified results and created coding trees for subsequent coding. Emerging themes were compared with constructs drawn from social cognitive theory, the health belief model, the theory of planned behaviour and reasoned action, the trans-theoretical model, the precaution adoption process model and the information-motivation-behavioural skills model, and coding also captured emerging pathways. Differences in perceived benefits between intervention and control arms were explored by comparing the proportion of participants reporting benefits to awareness and change in general and in the four targeted domains. The relationship between qualitative findings and survey data on goals and supports were explored using cross-tabulations. |
| Details of participants | Location- country (region): | United States (Los Angeles) |
|  | Target population: | People living with HIV |
|  | Sampling: | Participants were recruited via study flyers targeting clients at 2 AIDS service organisations. Eligible participants spoke English and reported taking medication daily; using alcohol, tobacco and/or drugs at least weekly; sexual activity at least weekly; and daily mobile phone and internet use. |
|  | Actual sample: | 50 participants consented and enrolled:   - Intervention group A (‘Assessment’): N=14 - Intervention group B (‘Behaviour Change’): N=20 - Control group C (bi-weekly Web survey only): N=16   Participation in qualitative interviews for process evaluation:   - Week 2   - 92.9% of intervention group A   - 85% of intervention group B   - 68.8% of control group C   - 82% overall - Week 4   - 85.7% of intervention group A   - 60% of intervention group B   - 56.3% of control group C   - 66% overall - Week 6   - 42.9% of intervention group A   - 30% of intervention group B   - 25% of control group C   - 32% overall |
|  | Sexuality: | - **Intervention group A:** 23.1% bisexual, 61.5% gay, 15.4% heterosexual - **Intervention group B:** 15% bisexual, 65% gay, 20% heterosexual - **Control group C:** 81.3% gay, 18.8% heterosexual |
|  | Gender identity: | - **Intervention group A:** 7.1% female, 78.6% male, 14.3% transgender - **Intervention group B:** 20% female, 75% male, 5% transgender - **Control group C:** 6.7% female, 93.3% male |
|  | Ethnicity: | - **Intervention group A:** 50% Black, 7.1% Latino, 28.6% White, 14.3% Mixed race - **Intervention group B:** 40% Black, 15% Latino, 5% Native American, 30% White, 10% Mixed race - **Control group C:** 56.3% Black, 25% Latino, 18.8% White |
|  | Socioeconomic status: | Not stated |
|  | Age: | Not stated |
| *Quality assessment* |  |  |
| *Questions used to judge rigour and relevance* | *Reviewer judgement* | *Description* |
| Were steps taken to minimise bias and error/increase rigour in sampling? | Y | Large sample recruited from two agencies |
| Were steps taken to minimise bias and error/increase rigour in data collection? | Y | Conducted qualitative interviews at three time-points and complemented with web survey data |
| Were steps taken to minimise bias and error/increase rigour in data analysis? | Y | Detailed description of analysis methods, which included coding by two independent researchers and checking by lead researcher |
| Were the findings of the study grounded in/supported by data? | Y | Key findings clearly supported by quotations |
| Was there good breadth and depth achieved in the findings? | Y | Range of topics explored with in-depth interpretation |
| The perspectives of men who have sex with men privileged? | Y | Conducted three waves of qualitative interviews; participants primarily male and gay or bisexual |
| *Overall reliability and usefulness of findings* | | |
| Reliability of findings | M | Rigorous data collection and analysis; however, interview response rates were low |
| Usefulness of findings | H | Provides useful findings on how receipt varied by patient characteristics |
| ***Programme:*** ***WRAPP*** *(Bowen 2007)^10^* | | |
| Methods | Overall study design: | RCT |
|  | Research questions/ hypotheses: | Not stated |
|  | Timing and duration: | Recruitment took place during April-May 2004. Following the pre-test and the intervention, participants completed the first post-test 7-14 days post-intervention and those in the intervention (not waitlist control) group then completed a follow-up assessment 7-14 days after the post-test. Process evaluation questions appear to have been asked at post-test. |
|  | Aspects of process evaluated: | Acceptability |
|  | Evaluates how processes vary by intervention characteristics, providers, participants and/or contexts? | Explored how receipt varied by characteristics of context |
|  | Data collection: | Four questions on intervention acceptability – asking about interest, usefulness, whether user would do the intervention again and whether they would recommend it to a friend – were answered using six-point Likert-type scales. A fifth question asked whether the time it took for pictures to load was just right, too short or too long. |
|  | Data analysis: | Analysis for process evaluation questions was not specified. Mann-Whitney *U* test was used to compare responses from users with dial-up internet connections to those with high-speed internet connections on item assessing acceptability of the length of time to load pictures. |
| Details of participants | Location- country (region): | United States (rural areas) |
|  | Target population: | Internet-using MSM living in rural areas |
|  | Sampling: | Participants were recruited face-to-face and via Internet banners at a popular website. Eligible participants were at least 18 years old, had sex with another man in the last 12 months and lived in a rural area. |
|  | Actual sample: | 90 men completed the pre-test and were randomised to intervention or waitlist control. 20% of intervention and 21% of waitlist control participants dropped out before completing all activities. Overall study completion was 78.9%; however, it appears that acceptability questions were asked prior to the last survey point. 74 participants (82%) responded to process evaluation questions. |
|  | Sexuality: | - **Intervention arm:** 92% gay, 8% bisexual/heterosexual - **Waitlist arm:** 91% gay, 9% bisexual/heterosexual |
|  | Gender identity: | Not stated |
|  | Ethnicity: | - **Intervention arm:** 23% non-White, 77% White - **Waitlist arm:** 16% non-White, 84% White |
|  | Socioeconomic status: | **Intervention arm**   - Employment: 68% full-time, 13% part-time/occasional, 18% not working - Income: under $15,000 (36%); $15,000-$24,999 (23%); $25,000-$49,999 (31%); > $50,000 (10%)   **Waitlist arm**   - Employment: 64% full-time, 20% part-time/occasional, 16% not working - Income: under $15,000 (37%); $15,000-$24,999 (31%); $25,000-$49,999 (20%); > $50,000 (12%) |
| *Quality assessment* | | |
| *Questions used to judge rigour and relevance* | *Reviewer judgement* | *Description* |
| Were steps taken to minimise bias and error/increase rigour in sampling? | Y | Process evaluation was conducted with full trial sample and response rate was good |
| Were steps taken to minimise bias and error/increase rigour in data collection? | N | Acceptability assessed using a few, fixed-response questions with no information provided on piloting or previous testing |
| Were steps taken to minimise bias and error/increase rigour in data analysis? | Y | Straightforward reporting of percentage and mean calculations; statistical tests are named where significance is presented |
| Were the findings of the study grounded in/supported by data? | Y | Sufficient data are presented to support findings and conclusions |
| Was there good breadth and depth achieved in the findings? | N | Explores acceptability using narrow range of questions which do not explore experiences with or views on specific aspects of the intervention |
| The perspectives of men who have sex with men privileged? | N | The data are from MSM but are based solely on a few, fixed-response items |
| *Overall reliability and usefulness of findings* | | |
| Reliability of findings | H | Data collected are narrow but likely to be valid; high response rate |
| Usefulness of findings | L | Very little information provided on how delivery/receipt varied |
| ***Programme:*** ***WRAPP*** *(Williams 2010)* | | |
| Methods | Overall study design: | Participants were randomised to 1 of 3 intervention module orders |
|  | Research questions/ hypotheses: | The study aimed to “assess how participants in an Internet HIV/AIDS health promotion intervention perceived the experience.”^(p.2)^ Specific objectives were to evaluate:   - if intervention completion varied by computer issues or satisfaction with intervention delivery - if satisfaction changed from completion of the fist module to completion of all three modules - if satisfaction was associated with the order in which modules were encountered |
|  | Timing and duration: | Baseline data were collected before randomisation. Post-module assessments were completed after module completion. Each module and its assessment had to be completed within 14-day period. |
|  | Aspects of process evaluated: | Reach, acceptability |
|  | Evaluates how processes vary by intervention characteristics, providers, participants and/or contexts? | Explored how receipt varied by context and by characteristics of participants and of the intervention |
|  | Data collection: | Baseline survey collected sociodemographic and computer-related data. Computer variables were measured by modem speed, computer location (home or public location) and time of day initially visiting the intervention. Satisfaction was assessed by intervention completion. Survey items after each module assessed technical aspects and acceptability, including: time to load screens (binary response), ease of navigating (5-point Likert-scale), acceptability of pictures and stories and of time to complete module activities (binary), interest in module activities and usefulness (low, moderate, high), and whether user would participate in intervention again and whether they would recommend it to a friend (yes/no). |
|  | Data analysis: | - Compared groups using chi-square, *t* tests and Mann Whitney test of proportions; significance set at *p* < 0.05. Where differences were significant, calculated odds ratios and 95% confidence intervals. - Explored differences in completion by sociodemographic variables, computer access and intervention satisfaction. Dropping out/failing to complete intervention was defined as completing only one module. - Among those completing all modules, examined significant changes after completing first and all 3 modules - Explored differences in interest in and reported usefulness of modules by module order, comparing hose encountering a module first and those encountering it last. |
| Details of participants | Location- country (region): | - United States (rural) |
|  | Target population: | Sexually active MSM in rural areas |
|  | Sampling: | Recruitment via banner ad on a popular MSM dating website. Eligible participants were male, aged 18 years or older, reported sex with another man in the past year and lived in a rural area at least an hour’s drive from a major urban area. |
|  | Actual sample: | 300 participants, of which 84% (N=252) completed the first and second modules and 73% (N=219) completed all 3 modules. |
|  | Sexuality: | - **Baseline:** 15% heterosexual/bisexual, 85% homosexual - **Completed:** 15% heterosexual/bisexual, 85% homosexual - **Dropped out:** 13% heterosexual/bisexual, 86% homosexual |
|  | Gender identity: | Not stated |
|  | Ethnicity: | - **Baseline:** 77% White, 3% African American, 10% Hispanic, 3% Asian, 5% Native American - **Completed:** 77% White, 2% African American, 10% Hispanic, 8% Asian, 4% Native American - **Dropped out:** 84% White, 3% African American, 10% Hispanic, 3% Asian, 1% Native American |
|  | Socioeconomic status: | **Baseline**   - Education: 31% less than high school, 12% high school, 57% college - Income: < $15,000 (39%); $15,000-$24,999 (25%); $25,000-$49,999 (27%); ≥ $50,000 (9%)   **Completed**   - Education: 4% less than high school, 15% high school, 82% college - Income: < $15,000 (34%); $15,000-$24,999 (28%); $25,000-$49,999 (27%); ≥ $50,000 (11%)   **Dropped out**   - Education: 3% less than high school, 17% high school, 80% college - Income: < $15,000 (49%); $15,000-$24,999 (14%); $25,000-$49,999 (26%); ≥ $50,000 (11%) |
|  | Age: | - **Baseline:** Age in years: 18-29 (67%), 30-39 (18%), >40 (15%) - **Completed:** Age in years: 18-29 (66%), 30-39 (21%), >40 (14%) - **Dropped out:** Age in years: 18-29 (66%), 30-39 (18%), >40 (15%) |
| *Quality assessment* | | |
| *Questions used to judge rigour and relevance* | *Reviewer judgement* | *Description* |
| Were steps taken to minimise bias and error/increase rigour in sampling? | Y | Large sample, purposively focused on sexually active, rural MSM |
| Were steps taken to minimise bias and error/increase rigour in data collection? | Y | Data collected via multiple questions and user completion data to assess engagement and acceptability |
| Were steps taken to minimise bias and error/increase rigour in data analysis? | Y | Data analysis methods are well-described |
| Were the findings of the study grounded in/supported by data? | Y | Findings are supported by quantitative data |
| Was there good breadth and depth achieved in the findings? | N | General findings presented on completion and satisfaction, but does not provide in-depth examination of reasons |
| The perspectives of men who have sex with men privileged? | N | Data come from MSM but are based on quantitative measures that do not allow participants to set out their own views |
| *Overall reliability and usefulness of findings* | | |
| Reliability of findings | H | Large sample, and research methods appear rigorous |
| Usefulness of findings | L | Findings on completion and acceptability are general and lack depth |

AIDS = acquired immunodeficiency virus

ANOVA = analysis of variance

CBT = cognitive behavioural therapy

HIV = human immunodeficiency virus

IMB Model = information-motivation-behavioural skills model

MSM = men who have sex with men

MTurk = Mechanical Turk

nPEP = non-occupational postexposure prophylaxis

PrEP = pre-exposure prophylaxis

RCT = randomised controlled trial

SD = standard deviation

SES = socioeconomic status

STI = sexually transmitted infection

## References

1. Sullivan P, Driggers R, Stekler J, et al. Usability and Acceptability of a Mobile Comprehensive HIV Prevention App for Men Who Have Sex With Men: A Pilot Study. *JMIR Mhealth Uhealth.* 2017;5(3):14.

2. Mustanski B, Garofalo R, Monahan C, Gratzer B, Andrews R. Feasibility, Acceptability, and Preliminary Efficacy of an Online HIV Prevention Program for Diverse Young Men who have Sex with Men: The Keep It Up! Intervention. *AIDS Behav.* 2013;17(9):14.

3. Greene G, Madkins K, Andrews K, Dispenza J, Mustanski B. Implementation and evaluation of the Keep it Up! online HIV prevention intervention in a community-based setting. *AIDS Education and Prevention.* 2016;28(3):15.

4. Madkins K, Moskowitz D, Moran K, Dellucci T, Mustanski B. Measuring Acceptability and Engagement of the Keep It Up! Internet-based HIV Prevention Randomized Controlled Trial for Young Men who have Sex with Men. *AIDS Educ Prev.* 2019;31(4):287-305.

5. Avellar T. *The feasibility and acceptability of an online mindfulness-based cognitive therapy intervention for same-sex attracted men*, University of California Santa Barbara; 2016.

6. Mustanski B, Greene G, Ryan D, Whitton S. Feasibility, Acceptability, and Initial Efficacy of an Online Sexual Health Promotion Program for LGBT Youth: The Queer Sex Ed Intervention. *The Journal of Sex Research.* 2015;42(2):11.

7. Lucassen M, Merry S, Simon Hatcher S, Frampton C. Rainbow SPARX: A Novel Approach to Addressing Depression in Sexual Minority Youth. *Cognitive and Behavioral Practice.* 2015;22(2):14.

8. Lucassen M, Hatcher S, Fleming T, Stasiak K, Shepherd M, Merry S. A qualitative study of sexual minority young people’s experiences of computerised therapy for depression. *Australasian Psychiatry.* 2015;23(3):6.

9. Swendeman D, Ramanathan C, Baetscher L, et al. Smartphone self-monitoring to support self-management among people living with HIV: Perceived benefits and theory of change from a mixed-methods, randomized pilot study. *J Acquir Immune Defic Syndr.* 2015;69:12.

10. Bowen A, Horvath K, Williams M. A randomized control trial of Internet-delivered HIV prevention targeting rural MSM. *Health Education Research.* 2007;22(1):8.
