# Supplementary material for: eHealth Interventions to Address Sexual Health, Substance Use, and Mental Health Among Men Who Have Sex With Men: Systematic Review and Synthesis of Process Evaluations
Source: J Med Internet Res. 2021 Apr 23;23(4):e22477. doi: 10.2196/22477 (PMC8105760; doi:10.2196/22477)
Supplement: Multimedia Appendix 4 [file jmir_v23i4e22477_app4.docx]

# Appendix 4. Coding structure for process evaluation synthesis

| **Primary codes** | **Secondary codes** | **Tertiary codes** |
| --- | --- | --- |
| HIV testing | Features | Intervention factors affecting variation in intervention receipt: Intervention features |
| nPEP |  |  |
| Ordering condoms |  |  |
| Ordering HIV test kits |  |  |
| PrEP content |  |  |
| Reminders |  |  |
| Boring | Content | Intervention factors affecting variation in intervention receipt: Barriers |
| Cheesy/strange |  |  |
| Content not enjoyable |  |  |
| Content suggestions |  |  |
| Content too difficult, or confusing |  |  |
| Content boring, common sense, repetitive |  |  |
| Intrusive |  |  |
| Too easy |  |  |
| Unclear |  |  |
| Media (-) | Format and presentation |  |
| Not enough media |  |  |
| Required additional materials |  |  |
| Want less talking and dialogue, more game play |  |  |
| Pacing | Length, pacing and time |  |
| Too busy to complete |  |  |
| Too long or slow |  |  |
| Age-inappropriate, e.g. ‘babied’ participants | Tailoring and applicability |  |
| For games, level of challenge inappropriate, e.g. for age |  |  |
| Limited value/relevance to own life |  |  |
| Not personal or tailored enough |  |  |
| Insufficiently gay-specific | Inappropriate orientation to gender or sexual identity/ behaviour |  |
| For interventions also targeting LGBTQ women, irrelevant content for men |  |  |
| Insufficient content for trans people |  |  |
| Not optimised for mobile | Technical |  |
| Technical aspects |  |  |
| Technical problems |  |  |
| Intrusive, too personal, privacy concerns |  |  |
| Language and terms, including gay stigmatising | Other barriers |  |
| Omitting key issues, e.g. PrEP |  |  |
| Approach | Content | Intervention factors affecting variation in intervention receipt: Facilitators |
| Content was clear, understandable and up-to-date |  |  |
| Doesn’t use scare tactics |  |  |
| Enjoyed content |  |  |
| Information, knowledge |  |  |
| Information not available elsewhere; about mental health; about broad sexual, emotional, relationship health and sexual function not just STIs |  |  |
| Intervention structure |  |  |
| Learning skills |  |  |
| Liked characters |  |  |
| Liked content, found it interesting |  |  |
| Not just information |  |  |
| Encouraged: adherence to a plan, communication/ closeness with partner | Mechanisms |  |
| Opportunities for reflection |  |  |
| Opportunities for self-expression |  |  |
| Reflection: own behaviour/risks |  |  |
| Reflection: Inter-relations between substance use, sexual health and/or substance use |  |  |
| Can do at home | Format |  |
| Characters |  |  |
| Format, interface |  |  |
| Interaction |  |  |
| Materials |  |  |
| Media pleasant/attractive; variety of media/formats; enjoyable/fun/interesting |  |  |
| Daily vs. less frequent self-monitoring; regularity valued for monitoring-based approach | Length, pacing and time |  |
| For games, liked approach of game relevant to real life |  |  |
| Length of modules/sections appropriate |  |  |
| Own pace, self-directed |  |  |
| Pacing appropriate |  |  |
| Characters relatable/like own friends | Tailoring and applicability |  |
| Realistic and relevant scenarios |  |  |
| Tailored to demographic |  |  |
| Tailored to individual |  |  |
| Easy to use, did not require technical assistance | Technical |  |
| Few said there were a few technical issues, few technical problems accessing content |  |  |
| Trusted that data were secure | Other facilitators |  |
| Tone and language, not patronising, balance between personal/colloquial and professional language |  |  |
| Age | Demographic characteristics | Participant factors affecting variation in intervention receipt |
| Region |  |  |
| Race/ethnicity |  |  |
| Education level |  |  |
| Intervention engagement, completion | Other personal characteristics |  |
| Level of ART adherence |  |  |
| Receiving external therapy |  |  |
| Internet speed | Internet speed | Contextual factors affecting variation in intervention receipt |
